# DNA methylation analysis of *NOTCH1* variants reveals the first episignature for non-syndromic congenital heart defects

**DOI:** 10.1186/s13073-025-01587-6

**Published:** 2026-01-07

**Authors:** Gregor Dombrowsky, Liselot van der Laan, Ananília Silva, Jeroen Breckpot, Enrique Audain, Anna Wilsdon, Michael A. Levy, Niels Vos, Marcel Mannens, Jiao Wang, Anjali Jain, Robert Lesurf, David Winlaw, Connie R. Bezzina, Mary Ann Thomas, Almuth Caliebe, Sabine Klaassen, Felix Berger, Sven Dittrich, Brigitte Stiller, Hashim Abdul-Khaliq, Ingo Dähnert, Frances Bu’Lock, Siobhan Loughna, J. David Brook, Seema Mital, Robert B. Russell, Thomas Pickardt, Ulrike Bauer, Hans-Heiner Kramer, Anselm Uebing, Peter Henneman, Bekim Sadikovic, Alex Postma, Marc-Phillip Hitz

**Affiliations:** 1 Present Address: Institute for Medical Genetics, Carl-von-Ossietzky-University, Oldenburg, Germany; 2 Present Address: Department of Human Genetics, Amsterdam Reproduction & Development Research Institute, Amsterdam University Medical Centers, University of Amsterdam, Amsterdam, The Netherlands; 3 Department of Pathology and Laboratory Medicine, Western University, London, ON Canada; 4 Center for Human Genetics, University Hospitals Leuven, Leuven, Belgium; 5 Department of Human Genetics, University of Leuven, KU Leuven, Leuven, Belgium; 6 School of Life Sciences, University of Nottingham, Nottingham, UK; 7 Verspeeten Clinical Genome Centre, London Health Sciences Centre, London, ON Canada; 8 Department of Human Genetics, Amsterdam Reproduction & Development Research Institute, Amsterdam University Medical Centers, University of Amsterdam, Amsterdam, The Netherlands; 9 BioQuant & Biochemistry Center, Heidelberg University, Heidelberg, Germany; 10 The Centre for Computational Medicine, The Hospital for Sick Children, Toronto, ON Canada; 11 Genetics and Genome Biology Program, The Hospital for Sick Children, Toronto, ON Canada; 12 Ann and Robert H. Lurie Children’s Hospital of Chicago and Feinberg School of Medicine, Northwestern University, Chicago, USA; 13 Department of Experimental Cardiology, Cardiomyopathy & Arrhythmia, Amsterdam Cardiovascular Sciences, Amsterdam UMC location University of Amsterdam, Amsterdam, the Netherlands; 14 Departments of Medical Genetics and Pediatrics, Cumming School of Medicine, University of Calgary, Calgary, AB Canada; 15 Institute of Human Genetics, University Hospital Schleswig-Holstein, University of Kiel, Kiel, Germany; 16 Experimental and Clinical Research Center (ECRC), Charité - Universitätsmedizin Berlin and Max Delbrück Center, Berlin, Germany; 17 Dept. of Congenital Heart Disease-Pediatric Cardiology, Deutsches Herzzentrum der Charité, Berlin, Germany; 18 Department of Pediatric Cardiology, University Hospital Erlangen, Friedrich-Alexander-University Erlangen-Nürnberg, Erlangen, Germany; 19 Department of Congenital Heart Disease and Pediatric Cardiology, University Heart Center Freiburg - Bad Krozingen, Medical Center - University of Freiburg, Freiburg, Germany; 20 Department of Pediatrics and Pediatric Intensive Care Medicine, University Hospital of Saarland, Homburg, Germany; 21 Department of Pediatric Cardiology, Heart Center Leipzig , University of Leipzig, Leipzig, Germany; 22 Congenital and Paediatric Cardiology, East Midlands Congenital Heart Centre, University of Leicester, Glenfield Hospital, Leicester, UK; 23 Division of Cardiology, Department of Pediatrics, The Hospital for Sick Children, University of Toronto, Toronto, ON Canada; 24 German Competence Network for Congenital Heart Defects, National Register for Congenital Heart Defects, Berlin, Germany; 25 Department of Congenital Heart Disease and Pediatric Cardiology, University Hospital of Schleswig-Holstein, Kiel, Germany; 26 DZHK (German Center for Cardiovascular Research), partner site University Hospital of Schleswig-Holstein, Kiel, Germany; 27 Department of Medical Biology, Amsterdam University Medical Centers, University of Amsterdam, Amsterdam, The Netherlands

**Keywords:** Congenital heart defects, NOTCH1, Disulfide-bridges, DNA-methylation, Tetralogy of Fallot, Episignatures

## Abstract

**Background:**

Congenital heart defects (CHDs) are the most common malformation amongst newborns, with a prevalence of approximately 0.8–2%. The etiology of CHD is highly complex and can be linked to genetic and nongenetic factors. The molecular basis remains partially unclear, and only a minority of patients can be assigned to clear monogenic causes.

**Methods:**

Here we analyzed a cohort of 3907 CHD cases and population-matched controls using exome sequencing. In addition, we employed epigenetic profiling on a subset of cases that harbored rare *NOTCH1* variants.

**Results:**

We identified 24 pathogenic or likely pathogenic single nucleotide variants (SNVs) in *NOTCH1* in our exome cohort, as well as a further 15 variants of uncertain significance (VUS) likely to have a deleterious effect. Although the cardiac phenotypes showed some heterogeneity, non-syndromic Tetralogy of Fallot (ToF) and related malformations were the most frequent finding in 56% (22/39). In particular, missense variants altering cysteine residues involved in forming disulfide bridges were identified, specifically in TOF patients. Altogether, *NOTCH1*-haploinsufficiency represented the most common monogenic cause in our cohort and accounted for an estimated 1% of CHD cases. Combined with additional cases assembled through collaborations, we present 67 individuals with ultrarare variants affecting *NOTCH1*.

This prominent role of *NOTCH1* calls for an accurate and accessible evaluation of variants. To this end we explored DNA methylation testing and successfully established a *NOTCH1*-specific episignature. This signature also displays a robust specificity in relation to 99 other episignatures. Taken together, we found that truncating, splice-altering, as well as missense *NOTCH1* variants, can generate a distinct DNAm episignature.

**Conclusions:**

We identified that *NOTCH1*-haploinsufficiency variants represented the most common monogenic cause in our cohort and accounted for an estimated 1 % of CHD cases. Furthermore, we conclude that methylation profiling can contribute to (NOTCH1) variant interpretation and improve the diagnostic management of CHD patients. Lastly, we established a *NOTCH1*-specific episignature, which represents the first non-syndromic signature, significantly extending the scope of patients that can benefit from methylation analysis.

**Supplementary Information:**

The online version contains supplementary material available at 10.1186/s13073-025-01587-6.

## Background

Congenital heart defects (CHD) are one of the most common birth abnormalities, affecting approximately 0.8–2% of live births worldwide [1–3]. It is widely accepted as having a multifactorial etiology with complex interactions between genetic and environmental factors during fetal development [4]. 

NOTCH1-signalling is one of the most important mechanisms during embryogenesis [5]. *NOTCH1* encodes a large single-pass membrane receptor that is involved in cell fate determination, differentiation, and the development of the nervous and cardiovascular systems. The latter includes regulation of cardiac precursor development, angiogenesis, vasculogenesis, and epithelial-mesenchymal transition during valve development [5, 6]. 

Initial reports of non-syndromic CHD in humans associated with *NOTCH1*-variants focused on malformations of the left ventricular outflow tracts (MIM#109730). However, subsequent publications have significantly broadened associated phenotypes, suggesting that conotruncal malformations of the cardiac outflow tract are a prevailing outcome, with phenotypes such as Tetralogy of Fallot (ToF), truncus arteriosus communis (TAC) and double outlet right ventricle (DORV) [7, 8]. Patients with *NOTCH1*-variants may have an elevated risk for aneurysms of the ascending aorta [9]. Somatic activating *NOTCH1* variants have also been related to tumorigenesis [10, 11]. In addition, *NOTCH1*-variants are known to cause Adams-Oliver syndrome, a condition mainly characterized by terminal transverse limb defects, aplasia cutis congenita, and various forms of CHD [12]. 

Understanding the genetic basis of CHD is crucial for improving diagnosis, for outcome or recurrence risk prediction, and for developing targeted therapies. Although next-generation sequencing (NGS) has been successful in identifying genetic variants associated with CHD, the mechanisms by which these pathogenic variants lead to CHD remain largely unknown. In addition, genomic studies are complicated by genetic heterogeneity of CHD and by the abundance of variants of unknown significance (VUS). Individual functional testing of VUSs to confirm or refute their contribution to CHD is complex and time-consuming, and typically not performed in a diagnostic context. Recently, the testing of episignatures (DNAm) has evolved as an easy method to screen such cases, as it uses readily available genomic DNA from peripheral blood samples.

Epigenetics involves the study of heritable changes in gene expression that occur without altering the underlying DNA sequence. Among these mechanisms, DNA methylation is the most thoroughly studied. Numerous rare genetic disorders have been linked to unique DNA methylation profiles, known as episignatures [13]. In recent years, episignatures have emerged as robust and reliable biomarkers, playing a crucial role in diagnosing congenital genetic disorders and reclassifying VUSs [14–18]. Their application in clinical diagnostic laboratories has demonstrated significant utility in providing diagnoses for patients with suspected rare genetic conditions who previously lacked a clear genetic diagnosis [19]. 

Reports of *de novo* variants histone modifying genes as well as altered DNA methylation in the context of CHD suggest that these mechanisms might contribute to the etiology of this disease [20–22]. 

Given that our cohort revealed *NOTCH1* as the most common monogenic cause, we aimed to explore whether effects of these variants might also manifest in the DNA-methylation pattern. In light of the high abundance of variants in *NOTCH1* in CHD patients, this episignature can have a considerable contribution to the diagnostic management of these patients.

## Methods

### Discovery cohort description

The work presented herein is primarily based on a cohort of 3907 exome-sequenced patients with CHD and 5157 population-matched controls [23, 24]. 1438 (37%) displayed extracardiac phenotypes (syndromic CHD, S-CHD), while 2469 (63%) non-syndromic heart defects (non-syndromic CHD, NS-CHD). 977 individuals presented with conotruncal defects, including 484 cases with ToF. Cases with various subgroups of CHD were included as long as the patient required intervention within the first year of life. Samples with known structural variations, such as 22q11 syndrome, or chromosomal aneuploidies, such as trisomy 21, were excluded if such a diagnosis was reported. Patient recruitment was conducted through multiple centers across Germany as well as from international centres [23, 24]. 

Samples were subjected to exome sequencing on DNA from peripheral blood using different versions of the SureSelect Exome chips (Agilent). Enriched libraries were subjected to 75-base paired-end sequencing (Illumina HiSeq). Data curation and quality filtering of the sequencing data were performed in accordance with previous work of our group [23, 24]. Samples were restricted to European ancestry.

Following the quality control steps outlined above, variants were functionally annotated using the Variant Effect Predictor tool (VEP v.104) [25], extended using the plug-ins CADD (version 1.6) and dbNSFP (version 4.1a) and evaluated based on the canonical transcript as defined by Ensembl (https://www.ensembl.org).

Functional domains and sites of post-translational modification in NOTCH1 were retrieved from UniProt (Identifier: P46531) (Table S1). Variants were collapsed into protein truncating variants (PTV), protein-altering variants (PAV) and synonymous variants (SYN).

Analyzed variants were prefiltered to an ultrarare frequency defined as a minor allele count (MAC) of ≤ 2 in gnomAD V4.1.0, in a set of internal unpublished control samples of German origin and the UK-BioBank (UKBB). Variant pathogenicity was assessed following the workflow presented by the American College of Medical Genetics (ACMG) [26, 27]. The severity of PAVs was assessed using in-silico prediction tools CADD, MPC, and REVEL [28–30]. Thresholds were used following the suggestions made by Pejaver et al. [31]

For the classification of splice site variants, SpliceAI was used with a cut-off of ≥ 0.5 [32]. Enrichment testing was carried out using a two-sided Fisher´s exact test (FET) and false-discovery rate (FDR) adjustment for multiple testing (*n* = 17 tests).

For all cases that underwent methylation profiling, genes related to DNA- or histone-methylation processes were reviewed. Genes were selected based on GO-terms GO:0035514, GO:0009008, GO:0140188, GO:0140940, GO:0140938 and GO:0140939 and filtered for species homo sapiens (see https://geneontology.org/) (Table S2). Variants were screened for pathogenic or likely pathogenic variants following the ACMG guidelines [26, 27]. 

### Assembly of additional cases

The findings from the initial cohort were extended using a genome-sequencing-based dataset of 1044 probands with non-syndromic congenital heart disease (CHD), containing 218 cases with transposition of the great arteries (TGA) and 826 with ToF. These samples were provided as part of a joint cohort from the Heart Centre Biobank Registry at the Hospital for Sick Children (Ontario, Canada), the Kids Heart BioBank at the Heart Centre for Children, The Children’s Hospital at Westmead (Sydney, Australia) and the CONCOR-project (Amsterdam Medical Center; Netherlands) [33, 34]. 

Sequencing was performed on DNA from blood or saliva of probands using the Illumina HiSeqX using the Illumina TruSeq DNA PCR-Free kit. The reads were trimmed and cleaned by trimmomatic v.0.32 [35], then mapped to human reference genome hg38 using bwa v.0.7.15 [36], followed by realignment and calibration(GATK v.4.1.2.0). HaplotypeCaller was used to generate genotype Variant Call Format (gVCF) files for each sample, combined and joint called (CombineGVCFs and GenotypeGVCFs tools). SNVs and indels were recalibrated separately by variant quality score recalibration (VQSR) tools, and variants that passed VQSR truth sensitivity level 99.5 for SNPs and level 99.0 for indels were retained. The VariantFiltration tool was used to mark out the low Genotype Quality (GQ) SNV and indel sites whose GQ values were lower than 20 and read depths were lower than 10.

Post processing of the data was performed using Bcftools view (v1.9) to subset the joint-called whole genome VCFs for the region of interest (chr9:136,484,054–136,580,643) [37], followed by decomposition and normalisation using vt v0.5, and annotation using VEP (v104.1) and VCFanno v0.3.1 [25, 38, 39]. 

Filtering and estimation of the deleteriousness of the variant were carried out as described for the initial cohort.

In addition, further variants were retrieved through personal communication with different collaboration partners, as outlined in Table 2.

### Review of published *NOTCH1*-variants

Variants in Clinvar and publications reporting *NOTCH1*-related cases were collected from PubMed as of June 2024. Search parameters were “NOTCH1 and CHD or congenital heart defects or AOS or Adams-Oliver syndrome”. Publications were manually revised. Variants that were explicitly cited from previous publications or with missing information regarding the position or patient’s phenotype were excluded, as were synonymous variants and variants that were considered benign by the authors (Table S3).

### Study cohort - methylation

A total of 26 individuals (12 males and 14 females) with *NOTCH1*-variants for whom material was available were included in the analysis of DNA methylation. The individuals were divided into one group for the discovery of the episignature (*n* = 19, of which 3 were negative for the episignature and removed) and additional samples to independently validate (*n* = 3) and assess VUS variants (*n* = 4). We used the discovery cohort for probe selection and construction of the classification model for the episignature. All of these individuals had confirmed deleterious variants in *NOTCH1*.

### DNA methylation data

Bisulfite-converted genomic DNA, extracted from peripheral blood, underwent application to the Infinium Methylation EPIC Bead Chip (Illumina, San Diego, CA) array following the manufacturer’s protocol. Subsequently, utilising the minfi R package (version 1.44.0) and the intensity data files (IDATS) containing methylated and unmethylated signal intensities produced post-EPIC array were preprocessed and imported into R (version 4.2.3) [40]. Standard preprocessing methods for Illumina microarrays were employed, involving background correction and normalisation. Quality control procedures included examining density plots and verifying concordance between recorded and predicted sex and age. Finally, probes were filtered by excluding probes overlapping with single-nucleotide variations, cross-reactive probes, probes specific to regions on the X or Y chromosomes, and probes with a detection p-value >0.1. The resulting number of probes after this filtration process was 772,557.

### DNA methylation analyses

DNA methylation analyses were conducted following our previously published methodology [13, 15]. Matched controls were chosen from the EpiSign Knowledge Database (EKD) based on age, sex, batch, and array type using the R package MatchIt (version 4.5.2) [41]. Samples exhibiting batch effects and/or more than 5% probe failure in the EKD were excluded. The training cohort and matched case-control samples underwent examination for data structure and outliers through principal component analyses (PCA). Subsequently, feature selection was performed using matched cases and controls. Differential methylation analysis was carried out utilizing the limma package (version 3.54.2) [42] with linear regression fitting. Methylation beta values served as predictors, and labels were used as the response, adjusting the model for estimated blood cell counts as confounding variables. The empirical Bayes method was applied to control for false discoveries, and adjustments were made using the Benjamini-Hochberg procedure to compute the moderated t-statistics and p-values. To ensure biological relevance, probes with a mean methylation difference below 5% (Δβ < 0.05) between cases and controls were excluded. Each remaining probe was ranked using a composite score that combined effect size (absolute Δβ) with statistical confidence (–log10 FDR-adjusted p-value). From this ranking, the top 800–1000 probes were retained. These were further refined by receiver operating characteristic (ROC) curve analysis (retaining probes with high AUC values) and by removing probes with high inter-probe correlation based on Pearson’s correlation coefficient, yielding a final set of 160–500 informative [13]. 

Further exploration involved investigating the distinct clustering of cases and controls using heatmaps and multidimensional scaling (MDS) with ggplots2 (version 3.1.3). The optimal clustering was selected based on parameter values. Leave-one-out cross-validation and unsupervised clustering results were employed to assess the reproducibility of the episignature (Figure S1).

### Prediction model

The sensitivity and specificity of the *NOTCH1*-episignature cohort were assessed through a classifier employing all episignature probes. A support vector machine (SVM) model was trained using the R package e1071 (version 1.7–13) with the selected features and matched controls and cases as training data. To enhance specificity, 75% of the samples in the EKD (comprising those with an episignature, unaffected samples, and training controls) were included, while the remaining 25% were designated for testing. This process was iterated four times, ensuring that each sample served as a testing sample once. The average SVM, also known as the methylation variant pathogenicity (MVP) score, was then employed for further analysis. Rare disease episignature classification typically involves a substantial proportion of unaffected or “normal” samples alongside affected cases. In this context, SVM’s provide a superior capacity compared to alternative machine learning models, as it allows more accurate discrimination between disease states and unaffected backgrounds, as well as among different episignature-positive conditions.

### Overlap of the *NOTCH1* genome-wide dna methylation profile with other episignature positive rare disorders

Functional annotation and comparison of the EpiSign™ classifier v5 cohort were conducted based on previously published articles [43]. The assessment involved determining the percentage of differentially methylated positions (DMPs) shared between the *NOTCH1*-episignature and the other 99 neurodevelopmental disorder episignatures on the EpiSign™ v5 clinical classifier. Heatmaps were created using the R package pheatmap (version 1.0.12), and circos plots were produced with the R package circlize (version 0.4.15) [44]. To identify relationships across all cohorts with known episignatures, clustering analysis was performed. Utilising the R package TreeAndLeaf (version 1.6.1) [45], a tree and leaf plot was generated to visualise the distances and similarities between the cohorts. For an exploration of the genomic location of the selected DMPs, probes were annotated in relation to CpG islands (CGIs) and genes using the R package annotatr (version 1.20.0) [46] with AnnotationHub (version 3.2.2), as described previously by Levy et al. [43]. 

### In-silico modelling of *NOTCH1* variants

For each variant-related region, structural models were generated using AlphaFold3. The modelled structures were subsequently subjected to conformational sampling with PyRosetta [47, 48], using 20 independent FastRelax trajectories under the ref2015 scoring function [49, 50]. Both backbone and side-chain flexibility were allowed, with disulfide bonds constrained according to the Uniprot annotations and AlphaFold3 prediction. Among the resulting models, the structures within the lowest energy were selected for downstream analysis. Underlying models and resulting structures are outlined in Table S4 and Figures S5-S9.

## Results

### Enrichment of deleterious *NOTCH1*-variants in a large CHD case-control cohort

In the analyzed cohort of 3907 exome-sequenced patients with CHD and 5157 population-matched controls, deleterious *NOTCH1*-variants were the most frequent monogenic finding. Filtering regarding ultrarare variants affecting the coding region and canonical splice sites of *NOTCH1* yielded 76 variants. Based on this initial variant set, enrichment testing was performed for truncating variants (PTVs), synonymous (SYN) and protein altering variants (PAVs), which were grouped based on in-silico predictions. Furthermore, distinct functional domains were tested for individual enrichment of PAVs. We investigated disulfide bridges, as these are frequent, especially in the extracellular EGF-like domains, and are essential for correct protein folding.

PTVs, as well as PAVs with strong in-silico pathogenicity predictions, were enriched (p_adj_ = 1.09e-04, p_adj_ = 0.047) (Table 1). Furthermore, ultrarare PAVs that disrupted disulfide bridges were enriched (p_adj_ = 0.025) and were almost exclusively found amongst patients with ToF (9/10 cases). In total, this type of variant was present in 1.85% (9/484) of ToF-cases. EGF-like repeats were also significantly more affected in CHD-cases. However, this was attributed mainly to overlap with disulfide-bond affecting variants (the exclusion of these variants results in a loss of significance (ratio 8 to 4, p_raw_ = 0.143)). Amongst the individual EGF-like repeats, no individual repeat was significantly enriched (data not shown). Interestingly, no PAVs in the vicinity of the ligand binding site (residues 420–421, 448–452, 469 each ± 5 residues) were observed. For none of the other tested functional domains, particular enrichment was observed. None of the discovered cases had pathogenic variants in established CHD genes or genes involved in DNA- or histone-methylation.

**Table 1:** Enrichment testing for ultrarare ***NOTCH1***-variants. Testing results of ultrarare variants affecting *NOTCH1* in 3907 CHD cases vs. 5157 controls. Testing was performed using FET. P-values were adjusted using false-discovery rate (FDR) with *n* = 17 tests. Significance was defined as p_adj_ < 0.05. Significant scenarios are printed in bold. PTVs are defined as stop-gain, frameshift, and splice-site variants. PAVs are defined as missense and indels. PP3 corresponds to the severity of in-silico prediction tools evaluating REVEL, CADD and MPC-score as proposed by Pejaver et al. [31] ANK = Ankyrin domain, CI = confidence interval, EGF = Epidermal growth factor, FDR = false discovery rate, HD = heterodimerisation domain, LNR = Lin12/Notch repeats, OR = odds ratio, PAV = Protein altering variant, PEST = PEST domain, PP3 = ACMG criterion for deleterious in-silico predictor, PSEN = Interaction with presenelin 1, PTV = Protein truncating variant, RAM = RBP-Jκ-associated module, TAD = transcriptional activation domain, SYN = synonymous variant

Scenario	Carrier cases	Carrier controls	p__raw_	p__FDR_	OR	CI95%
PTVs	17	1	6.41e-06	1.09e-04	22.53	3.5–937.8
Disulfide bonds	10	1	1.47e-03	0.025	13.23	1.9–572.8
EGF-like repeats	17	5	1.82e-03	0.030	4.50	1.6–15.6
PAVs (PP3str)	7	0	2.76e-03	0.047	Inf	1.9 - Inf
PAVs (PP3mod)	10	3	0.021	0.364	4.41	1.1–24.9
Novel cysteine formation	4	0	0.034	0.586	Inf	0.9 – Inf
EGF-like repeats (excl. Disulfide bonds)	8	4	0.143	1	2.64	0.7–12.0
RAM	1	0	0.431	1	Inf	0.03 - Inf
Ankyrin	2	1	0.581	1	2.64	0.1–155.7
PAVs (PP3sup)	3	2	0.658	1	1.98	0.2–23.7
SYN	6	6	0.772	1	1.32	0.4–4.9
TAD	3	3	1	1	1.32	0.2–9.9
LNR	2	2	1	1	1.32	0.1–18.2
PEST	2	2	1	1	1.32	0.1–18.2
PAVs (neutral or benign in-silico)	9	12	1	1	0.99	0.4–2.6
HD	0	1	1	1	0	0–51.4
PSEN	0	1	1	1	0	0–51.4

### Review of published *NOTCH1*-variants

Through a review of publications that reported *NOTCH1* variants, we assembled a list of 204 unique variants reported in 304 cases (Table S3). Of these cases, 238 were reported in the context of CHD, 22 in the context of thoracic aortic aneurysms (TAAD) and 44 in Adams-Oliver syndrome (AOS).

Missense variants were distributed throughout the entire protein without overrepresentation of particular domains. *NOTCH1* missense variants affecting disulfide bridges were reported in both CHD and AOS cases with a slight enrichment in AOS (14/238 CHD, 10/44 AOS, *p* = 0.001; OR 4.7, 95%-CI 1.71–12.4, two-sided FET). One disulfide-altering variant was also found in a TAAD-case. Interestingly, all 14 variants affecting disulfide bridges identified in CHD cases were reported to have ToF or related malformations. A comprehensive overview of all disulfide-impacting variants can be found in Table S5. In addition, PAVs in the vicinity of the ligand binding site were found in 8/44 AOS patients versus 2/238 CHD cases (*p* = 6.93e-06; OR 25.7, 95%-CI 4.9–256.5.9.5, two-sided FET).

### Assembly of additional cases

**Fig. 1:**
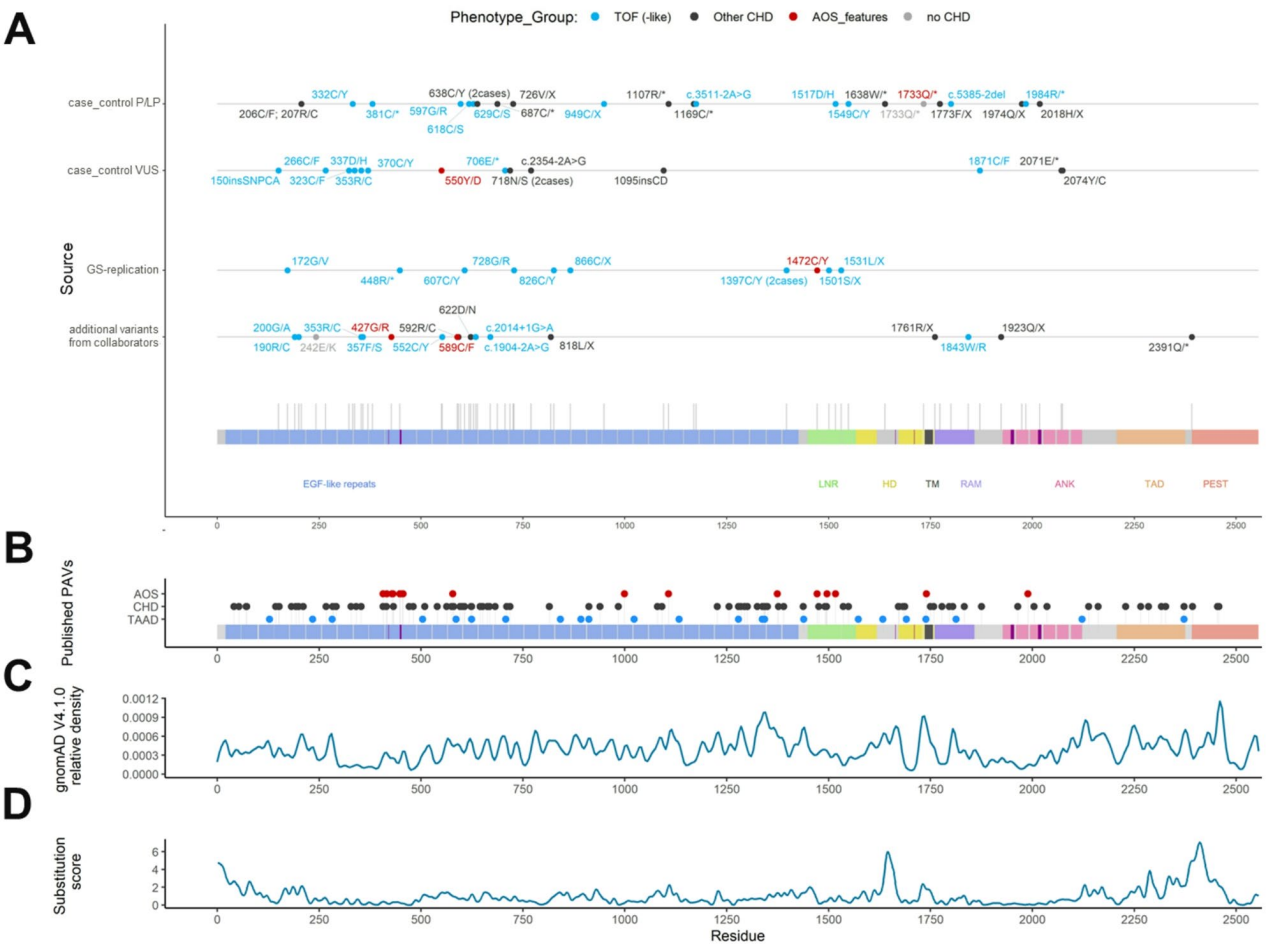
Overview of variants found in NOTCH1.** A**: Representation of *NOTCH1* variants found in the analyzed samples. Each dot represents an identified variant colour-coded by the corresponding phenotype group. “TOF (-like)” includes ToF, DORV and TAC (blue). “AOS_features” marks cases that displayed extracardiac anomalies possibly consistent with an AOS phenotype (red). Other CHD (darkgray) reprents all non-TOF-like phenotypes. Cases without CHD are shown in light gray. Functional domains are shown based on Uniprot (for details refer to Table S1). **B**: Overview of previously reported missense-variants. (see Table S3 for details). Variants are split and color-coded based on the corresponding reported phenotype. Black: (ns-)CHD, Red: Adams-Oliver-Syndrome, Blue: thoracic aortic aneuyrism. **C**: Density plot of missense variants present in gnomAD V4.1.0. **D**: Amino acid conservation as retrieved from Aminode.[53] Depicted is the substitution score per amino acid. High values indicate a low conservation of the residue

In an independent genome-sequenced cohort [34], we identified no variants similar to the ones in our case-control cohort among 218 cases of TGA. However, in 826 ToF cases, we identified four ultrarare PTVs, five cases with ultrarare PAVs disrupting disulfide bonds, and two additional ultrarare PAVs with strong in-silico prediction scores (Table 2). Collectively, deleterious variants in *NOTCH1* were thus found in 1.5% (10/641) of European ToF cases and 1.3% (11/839) of samples regardless of population (Fig. 1A). In addition, we assembled a further 17 samples from the centres of various co-authors. These either fulfilled the filtering criteria established above, were deemed potentially causal due to evidence from segregation with the disease within the family, or were considered of interest for validation purposes regarding the specificity of the episignature analysis (see below). Collectively, we found 63 ultrarare, deleterious *NOTCH1-*variants in 67 individuals (Fig. 1; Table 2).

**Table 2:** Overview of all identified *NOTCH1*-variants. Samples with pathogenic or likely pathogenic variants from the initial case-control cohort are listed, followed by samples with VUSs from the initial cohort. Lastly, variants from the GS-cohort and additional collaboration partners are presented. Variants are classified based on the ACMG-classification scheme. Where available, the segregation of the variant is given with [S] indicating, that a targeted testing was performed using Sanger-sequencing and [ES] indicating that the inheritance pattern was determined based on exome-sequencing data. ACH = Alberta Children´s Hospital, ASD = atrial septal defect, ASDII = secundum atrial septal defect, AUMC = Amsterdam University Medical Centre, AV= Aortic valve, BAV = bicuspid aortic valve, CCHMC = Cincinnati Children’s Hospital Medical Center, CoA = Coarctation of the aorta, DORV = double outlet right ventricle, F = female, HLHS = Hypoplastic left heart syndrome, HSC = Hospital for Sick Children Toronto, LP = likely pathogenic, LVOTO = Left ventricular outflow tract obstruction, M = male, MVP = Mitral valve prolapse, n.a. = not available, n.t. = not tested, P = pathogenic, PA = pulmonary artery atresia, PAH = Pulmonary artery hypertension, pDA = Patent ductus arteriosus, PvA = Pulmonary valve atresia, Pro = Proceed-cohort (replication GS-cohort), PS= Pulmonary stenosis, RV = right ventricle, TAC = Truncus arteriosus communis, TGA = Transposition of the great arteries, ToF = Tetralogy of Fallot, UHL = University Hospitals Leuven, VSD = ventricular septal defect, VUS = Variant of uncertain significance

Pathogenic or likely pathogenic variants										
#	NOTCH1 genotype (ref: NM_017617.5)	Class (ACMG)	Previously reported	Segregation status	Sample	Gender	Cardiac phenotype	Extacardiac phenotype	Familial CHD history	Remarks
1	c.617G>T p.(Cys206Phe); c.619C>T p.(Arg207Cys)	LP; VUS	Novel; ClinvarID: 2164530 (3x VUS)	n.t.	C08660	M	Hemitruncus	Anisocoria	n.a.	Positive DNAm signature (0.98549) [Discovery]
2	c.995G>A p.(Cys332Tyr); c.6376G>A p.(Gly2126Arg)	LP; VUS	COSM3716156; rs572960572	n.t.	C07803	M	ToF and variants	Seizure with abnormal EEG, neurodermitis, polyps as infant	Mother with TOF	Positive DNAm signature (0.98568) [Discovery]
3	c.1143T>A p.(Cys381Ter)	LP	Novel	n.t.	C08987	F	DORV	Osteoporosis, liver insufficiency, kidney insufficiency	Empty	Positive DNAm signature (0.98551) [Discovery]
4	c.1789G>C p.(Gly597Arg)	LP	Novel	n.t.	C08017	M	ToF and variants, dextrocardia	Hepatomegaly	Empty	Also carries VUS in *TLL1* (NM_012464.5:c.555G>C)
5	c.1852T>A p.(Cys618Ser)	LP	Novel	n.t.	C08732	M	ToF and variants	n.a.	Empty	
6	c.1886G>C p.(Cys629Ser)	LP	Novel	n.t.	C07885	F	ToF and variants, Aortic aneurysm	Hypothyroidism	n.a.	
7	c.1913G>A p.(Cys638Tyr)	LP	COSV53094192	n.t.	C08084	M	VSD	Neck fistula	n.a.	Father of C08085
8	c.1913G>A p.(Cys638Tyr)	LP	COSV53094192	Inherited from affected father [ES]	C08085	F	ToF and variants	Generalized muscular hypotonia	Father with VSD; Cousin with severe developmental delay; maternal uncle with spontaneously closed VSD	Daughter of C08084
9	c.2061C>A p.(Cys687Ter)	LP	Novel	n.t.	C08345	F	VSD	Recurrent infections, mild developmental delay	n.a.	Positive DNAm signature (0.9855) [Discovery]
10	c.2176_2181 GTGGAC>TGCAACA p.(Val726fs*16)	LP	Novel	*De novo* [S]	C07635	F	Hypoplastic right heart	n.a.	n.a.	Positive DNAm signature (0.98549) [Discovery]
11	c.2846_2856 GTGCCAGTGAC>CCT p.(Cys949Serfs*29)	LP	Novel	*De novo* [S]	C07558	M	ToF and variants	n.a.	n.a.	Positive DNAm signature (0.98548) [Discovery]
12	c.3319C>T p.(Arg1107Ter)	P	rs41309764, ClinvarID: 12476 (3x P), CM053346, PMID: 16025100	n.t.	C07810	F	Tricuspid atresia	Mild developmental delay	n.a.	Positive DNAm signature (0.98552) [Discovery]
13	c.3507C>A p.(Cys1169Ter)	LP	Novel	n.t.	C07994	M	HLHS	Hydrocephalus internus, mild muscular hypotonia, intracerebral haemorrhage, delayed wound healing, increased scarring, recurring otitis media	Empty	Positive DNAm signature (0.98548) [Discovery]
14	c.3511-2A>G p.(?)	LP	PMID: 26820064	*De novo* [S]	C07964	F	TAC, peripheral PS	Strabism, unilateral hearing impairment	Empty	Positive DNAm signature (0.98551) [Discovery]
15	c.4549G>C p.(Asp1517His)	LP	COSM124799, Clinvar: 3048375 (1x LP)	n.t.	C08171	F	ToF and variants	Hypothyroidism, kidney cyst, kidney insufficiency	n.a.	Positive DNAm signature (0.98549) [VUS-evaluation]
16	c.4646G>A p.(Cys1549Tyr)	LP	Novel	*De novo* [ES]	C01022	F	ToF and variants	n.a.	n.a.	
17	c.4913G>A p.(Trp1638Ter)	LP	Novel	n.t.	C00902	M	Abnormality of cardiovascular system morphology	Hemiatrophy, dentiogenesis imperfecta, inflammation of the large intestine, generalized joint laxity	n.a.	
18	c.5197C>T p.(Gln1733Ter); c.452A>G p.(Asn151Ser); c.3880G>A p.(Glu1294Lys)	LP; VUS; VUS	rs1208976166; rs766362765; COSM5880101, rs1247035429; PMID: 30582441	n.t.	C02192	M	ToF and variants	Craniosynostosis	Brother with aortic anomaly, maternal aunt with heart murmur	Positive DNAm signature (0.986) [Discovery]
19	c.5197C>T p.(Gln1733Ter); c.3880G>A p.(Glu1294Lys)	LP; VUS; VUS	rs1208976166; COSM5880101, rs1247035429; PMID: 30582441	n.t.	C02193	F	Control sample	n.a.	n.a.	
20	c.5315_5316G>GC p.(Phe1773fs*5)	LP	Novel	*De novo* [S]	C07685	F	Hypoplastic right heart	Recurring otitis media, hypertrophic osteoarthropathy	Empty	Positive DNAm signature (0.98551) [Discovery]
21	c.5385-2del p.(?)	LP	COSM5622683	*De novo* [ES]	C01099	M	ToF and variants	n.a.	n.a.	
22	c.5922CT>C p.(Gln1974fs*6)	LP	Novel	*De novo* [S]	C08838	F	ASDII	n.a.	n.a.	Positive DNAm signature (0.98549) [Discovery]
23	c.5950C>T p.(Arg1984Ter)	P	PMID: 26820064	n.t.	C08330	F	DORV	Liver insufficiency	n.a.	Positive DNAm signature (0.9855) [Discovery]
24	c.6053_6054del p.(His2018fs*7)	LP	Novel	n.t.	C07725	F	CoA, BAV, right PA	Hallux, scoliosis, recurrent severe infections, muscular hypotonia	Father with cardiac phenotype as a child (no details available)	Positive DNAm signature (0.98864) [Discovery]

Ultrarare variants of uncertain significance										
#	NOTCH1 genotype (ref: NM_017617.5)	Class (ACMG)	Previously reported	Segregation status	Sample	Gender	Cardiac phenotype	Extacardiac phenotype	Familial CHD history	Remarks
25	c.436_450dup p.(150_151ins SerAsnProCysAla)	VUS	ClinvarID: 2169548 (1x VUS)	n.t.	C07149	M	ToF and variants	n.a.	n.a.	Also carries VUS in *SMAD6* (NM_005585.5: c.511G>A) Creates novel cysteine Positive DNAm signature (0.98547) [Discovery]
26	c.797G>T p.(Cys266Phe) c.798C>T p.(Cys266=)	VUS; VUS	COSM6981052	Maternal [S]	C08918	F	ToF and variants	n.a.	n.a.	Predicted splice donor gain
27	c.968G>T p.(Cys323Phe)	VUS	COSM6903534	Paternal [S]	C08178	M	ToF and variants	n.a.	n.a.	Disrupts disulfide bridge
28	c.1009G>C p.(Asp337His)	VUS	Novel	n.t.	C08917	M	ToF and variants	n.a.	n.a.	Moderate *in-silico* score
29	c.1057C>T p.(Arg353Cys)	VUS	rs1300110216, PMID: 30582441	n.t.	C07205	F	ToF and variants	n.a.	n.a.	Creates novel cysteine
30	c.1109G>A p.(Cys370Tyr)	VUS	rs1564199987, ClinvarID: 576792 (1x VUS)	Maternal [ES]	C02162	F	ToF and variants	n.a.	n.a.	Disrupts disulfide bridge
31	c.1648T>G p.(Tyr550Asp)	VUS	COSM753122, ClinvarID: 1033432 (1x VUS)	n.t.	C06994	M	TAC	Facial asymmetry, sprengel anomaly, specific learning disability, high anterior hairline, plagiocephaly, micropenis, sleep disturbance, attention deficit hyperactivity disorder	n.a.	Moderate *in-silico* score
32	c.2116G>T p.(Glu706Ter)	VUS	Novel	Paternal [S]	C07611	M	DORV	Hepatomegaly	Empty	Truncating variant
33	c.2153A>G p.(Asn718Ser)	VUS	COSM4411745, ClinvarID: 1029477 (1xLP, 2x VUS), PMID: 31813956	n.t.	C00994	M	CoA	Autistic behaviour, delayed speech and language development, intellectual disability (mild), motor delay	n.a.	Predicted splice donor gain, brother of C01007
34	c.2153A>G p.(Asn718Ser)	VUS	COSM4411745, ClinvarID: 1029477 (1xLP, 2x VUS), PMID: 31813956	n.t.	C01007	M	VSD	Autism, delayed speech and language development, intellectual disability, (mild), poor coordination, moderate global developmental delay	n.a.	Predicted splice donor gain, brother of C00994
35	c.2354-2A>G p.(?)	VUS	COSV53109619	n.t.	C01673	M	HLHS	n.a.	n.a.	Altered splicing potentially preserves reading frame; Mild DNAm signature (0.88909)
36	c.3280_3285dup p.(1095_1096insCysAsp)	VUS	Novel	n.t.	C00068	M	Abnormal heart morphology	Microcephaly, congenital cataract, mixed hearing impairment, sleep apnoea, constipation	n.a.	Creates novel cysteine
37	c.5612G>T p.(Cys1871Phe)	VUS	Clinvar: 2182606 (2xVUS)	n.t.	C07622	M	ToF and variants	n.a.	n.a.	Affects cysteine residue, residue is not known to form a disulfide bond
38	c.6211del p.(Glu2071fs*38); c.2054A>C p.(Asn685Thr)	VUS; VUS	Novel; rs781473342	n.t.	C01759	M	CoA, BAV	n.a.	n.a.	Not predicted to undergo NMD, Positive DNAm signature (0.98551) [Discovery]
39	c.6221A>G p.(Tyr2074Cys)	VUS	Novel	Paternal [S]	C08510	M	CoA, Aneurysm fossa ovalis	Esophagus atresia, polyhydramnion	n.a.	Creates novel cysteine residue Positive DNAm signature (0.89228) [VUS-evaluation]

Variants from GS cohort										
#	NOTCH1 genotype (ref: NM_017617.5)	Class (ACMG)	Previously reported	Segregation status	Sample	Gender	Cardiac phenotype	Extacardiac phenotype	Familial CHD history	Remarks
40	c.515G>T p.(Gly172Val)	LP	novel	n.t.	Pro1	F	ToF and variants	No	Pat. grandfather and pat. first cousin with TOF	
41	c.1342C>T p.(Arg448Ter)	LP	rs869025494, PMID: 30582441	n.t.	Pro2	M	ToF and variants	Short sightedness	Pat. first cousin with CoA, MVP and small left ventricle	
42	c.1820G>A p.(Cys607Tyr)	LP	PMID 30582441	n.a.	Pro3	F	ToF and variants	No	Father with MVP	
43	c.2182G>C p.(Gly728Arg)	LP	novel	n.a.	Pro4	M	ToF and variants	No	Empty	Sample is predicted to be of East-asian ancestry
44	c.2477G>A p.(Cys826Tyr)	VUS	novel	Maternal	Pro5	M	ToF and variants	No	Empty	Disrupts disulfide-bridge
45	c.2595del p.(Cys866Valfs*10)	LP	novel	n.t.	Pro6	M	ToF and variants	No	Empty	
46	c.4190G>A p.(Cys1397Tyr)	LP	novel	n.t.	Pro7	F	ToF and variants	No	Unknown	
47	c.4190G>A p.(Cys1397Tyr)	LP	novel	n.t.	Pro8	M	ToF and variants	No	Unknown	
48	c.4415G>A p.(Cys1472Tyr)	LP	PMIDs: 30293987, 31813956	n.t.	Pro9	F	ToF and variants	Cutis aplasia	Nephew with TOF	
49	c.4501del p.(Ser1501Valfs*79)	LP	novel	n.t.	Pro10	M	ToF and variants	No	Mat. aunt with TOF, died w/o surgery at 6 m of age, Pat. grandfather has an MI at age of 60	
50	c.4592del p.(Leu1531Argfs*49)	LP	novel	n.t.	Pro11	F	ToF and variants	No	Unknown	

Additional variants from collaboration partners										
#	NOTCH1 genotype (ref: NM_017617.5)	Class (ACMG)	Previously reported	Segregation status	Sample	Gender	Cardiac phenotype	Extacardiac phenotype	Familial CHD history	Remarks
51	c.568C>T p.(Arg190Cys)	VUS		n.t.	Pro12	F	ToF and variants	Unknown	Unknown	Creates novel cysteine residue
52	c.599G>C p.(Gly200Ala)	VUS	novel	n.t.	Pro13	F	ToF and variants	Unknown	Unknown	Moderate *in-silico* prediction for pathogenicity
53	c.724G>A p.(Glu242Lys)	VUS	rs564629053	Inherited from affected mother	AUMC1	M	Cardiac evaluation: normal	Obesity, autism spectrum disorder, intellectual disability, alopecia, delayed puberty	Mother with similar phenotype	Negative for NOTCH1-DNAm signature (0.0157) [VUS-evaluation]
54	c.1057C>T p.(Arg353Cys)	VUS	PMID: 30582441	n.t	Pro14	F	ToF and variants	No	Mother's maternal cousin was born with CHD and died at a few days of age	Creates novel cysteine residue
55	c.1070T>C p.(Phe357Ser)	VUS	novel	Inherited from affected father	Pro15	F	ToF and variants	Microcephaly	Father - VSD Repair and Implantation of mechanical prosthetic AV	Moderate *in-silico* prediction for pathogenicity
56	c.1279G>C p.(Gly427Arg)	LP	novel	*De novo*	LUV1 (UHL)	F	Primary pulm. hypertension, pDA	Adams-Oliver Syndrome, alopecia, mild hand/feet anomalies, thrombopenia, granuloma of the upper lip	Empty	Negative for NOTCH1-DNAm signature (0.10763)
57	c.1655G>A p.(Cys552Tyr)	LP	novel	*De novo*	LUV2 (UHL)	M	ToF and variants	Plagiocephaly, delayed development (total IQ 83), ASS, long triangular face	Empty	Positive DNAm signature (0.98549) [Validation]
58	c.1774C>T p.(Arg592Cys)	VUS	rs1472690723	n.a.	AUMC2	M	TGA	Unknown	Unknown	MAC 17 in gnomAD V4.1.0 Negative for NOTCH1-DNAm signature (0.45762)
59	c.1766G>T p.(Cys589Phe)	LP	novel	n.a.	MT1 (ACH)	F	Mild PVS, not clinically significant	Height slightly < p3, Cutis aplasia, 6.5 cm long and 1 cm wide and at the midline top of the scalp, normal digits, possible subtle cutis marmorata, no brachydactyly	Two pregnancies with similar cardiac anomalies (1x ToF and variants, 1x DORV)	
60	c.1864G>A, p.(Asp622Asn)	VUS	rs367873715, Clinvar 659037	n.a.	AUMC3	M	Slight RV dilatation, Idiopathic PAH	Diabetes	Empty	Known PAH genes have been excluded, Negative for NOTCH1-DNAm signature (0.53211) [VUS-evaluation]
61	c.1904-2A>G p.(?)	VUS	novel	n.a.	DW1 (CCHMC)	F	ToF and variants	Choanal atresia	Father with small hole in heart	Not predicted to disrupt reading frame
62	c.2014+1G>A p.(?)	P	rs515726232, Clinvar 139664, PMID: 25931334	Inherited from affected father	LUV5 (UHL)	M	DORV, TGA	No	Father with ASDII, two siblings (fetuses) with HLHS	Positive DNAm signature (0.98551) [Validation]
63	c.2433_2452dup p.(Leu818Profs*65)	P	novel	Inherited from affected mother	JB1 (UHL)	M	CoA, asymmetric tricuspid AV, parachute-like MV	No	Mother with BAV, congenital strabism and ptosis of the right eyelid, Mat. grandfather with aneurysm of the abdominal aorta, he´s not carrier of the variant. Mat. grandmother had AV-replacement (no genetic testing)	
64	c.5281delC p.(Arg1761Glyfs*37)	P	rs515726231, PMID: 25931334	Inherited from affected father	LUV4 (UHL)	F	Mild CoA, asymmetric tricuspid AV	No	Father with CoA, paternal half-brother with ToF, PA, VSD, type DORV, several other family members with LVOTO, or non-affected carriers	Positive DNAm signature (0.9855) [Validation]
65	c.5527T>C p.(Trp1843Arg)	VUS	novel	n.t.	Pro16	M	ToF and variants	No	Unknown	Moderate *in-silico* prediction for pathogenicity
66	c.5767del p.(Gln1923fs*67)	P	novel	Inherited from affected father	SM1 (HSC)	M	HLHS/DORV	No	Father with PA & VSD, pat. uncle with ToF and variants, pat. grandmother with unspecified valve defect.	
67	c.7171C>T p.(Gln2391Ter)	VUS	novel	n.t.	SM2 (HSC)	M	HLHS	Developmental delay, speech disorder, hemiparesis right-sided	Empty	Not predicted to undergo nonsense-mediated decay

Of note, regions surrounding and preceding the extracellular ligand binding site, as well as the intercellular ankyrin-repeats, display high conservation and low variant abundance in the population. Interestingly, we did not observe variants near the ligand binding site (Fig. 1A), while this appears to be a region frequently affected in AOS (Fig. 1B).

### Discovery and validation of the *NOTCH1*-episignature

**Fig. 2:**
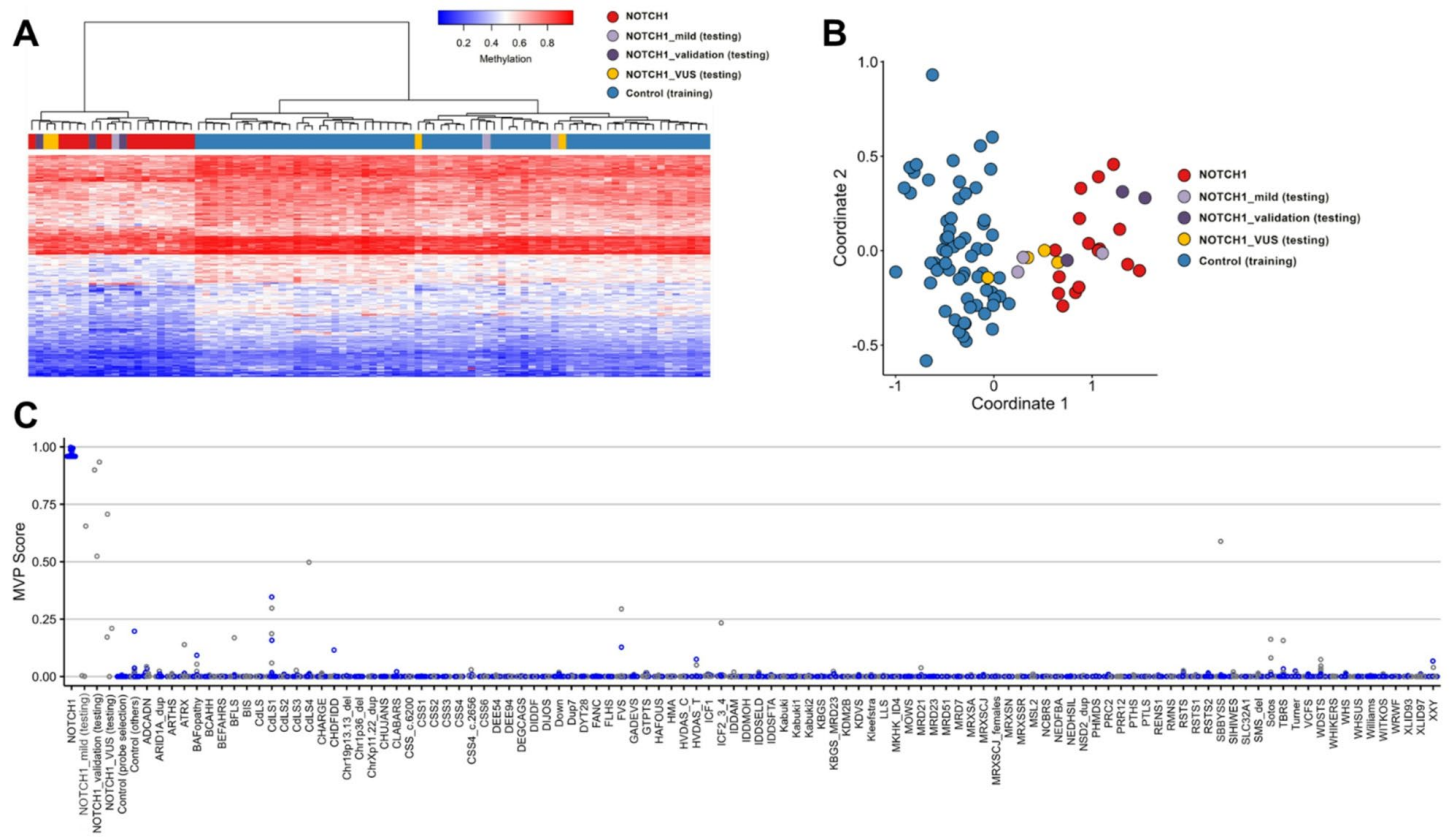
Discovery and validation of the ***NOTCH1***-episignature**. A**: The Euclidean hierarchical clustering heatmap depicts each column representing one NOTCH1 discovery case (highlighted in red), along with mild signatures (light purple), VUS (yellow), and validation samples (dark purple). Each row corresponds to a specific probe selected for this episignature. Notably, a distinct separation is observed between the cases (in red) and controls (in blue). However, it’s worth mentioning that the negative cases tend to cluster together with the controls, except for one. **B**: The multidimensional scaling (MDS) plot illustrates the separation between NOTCH1 cases and controls, including the negative sample identified in (A). **C**: In the SVM classifier model, the selected *NOTCH1*-episignature probes were used to train the model. 75% of controls and 75% of samples from other neurodevelopmental disorders (depicted in blue) were utilised for training, while the remaining 25% of controls and 25% of other disorder samples (grey) were reserved for testing

The collected cases displayed a heterogeneous phenotypic outcome as well as the type and classification of variants affecting *NOTCH1* which called for an accurate and accessible evaluation of these variants. We thus explored DNA methylation testing on a subset of available samples. The resultant probe set generated from a discovery cohort of 19 samples effectively distinguished between cases and controls (Fig. 2, Table S6). However, three samples from the discovery group (cases #35, #56, #58) didn’t align with the episignature and were excluded from the training set (Fig. 2, light purple). Two of these samples consistently grouped with controls in the heatmap and MDS plots, displaying MVP scores close to 0; the other sample had a higher MVP score (0.88) but was not consistently grouped with the discovery cases and couldn’t be included in the discovery cohort. To validate the *NOTCH1-*episignature, we used three additional samples with confirmed pathogenic variants in *NOTCH1* (cases #57, #62, #64), all of which aligned with the episignature (Fig. 2A, dark purple). Furthermore, each of these cases yielded a high MVP score, affirming their resemblance to our *NOTCH1-*episignature (Fig. 2B, dark purple).

After having successfully established an episignature, we investigated whether we could use it to reclassify VUSs. To this end, we evaluated four VUS samples (cases #15, #39, #53, #60). Our analysis revealed that two samples (cases #15, #39) aligned with the *NOTCH1-*episignature, while the other two did not (Fig. 2A and B, yellow).

Comparison of the *NOTCH1* global DNAm profile with other neurodevelopmental disorders included in the EpiSign V5 classifier.

**Fig. 3:**
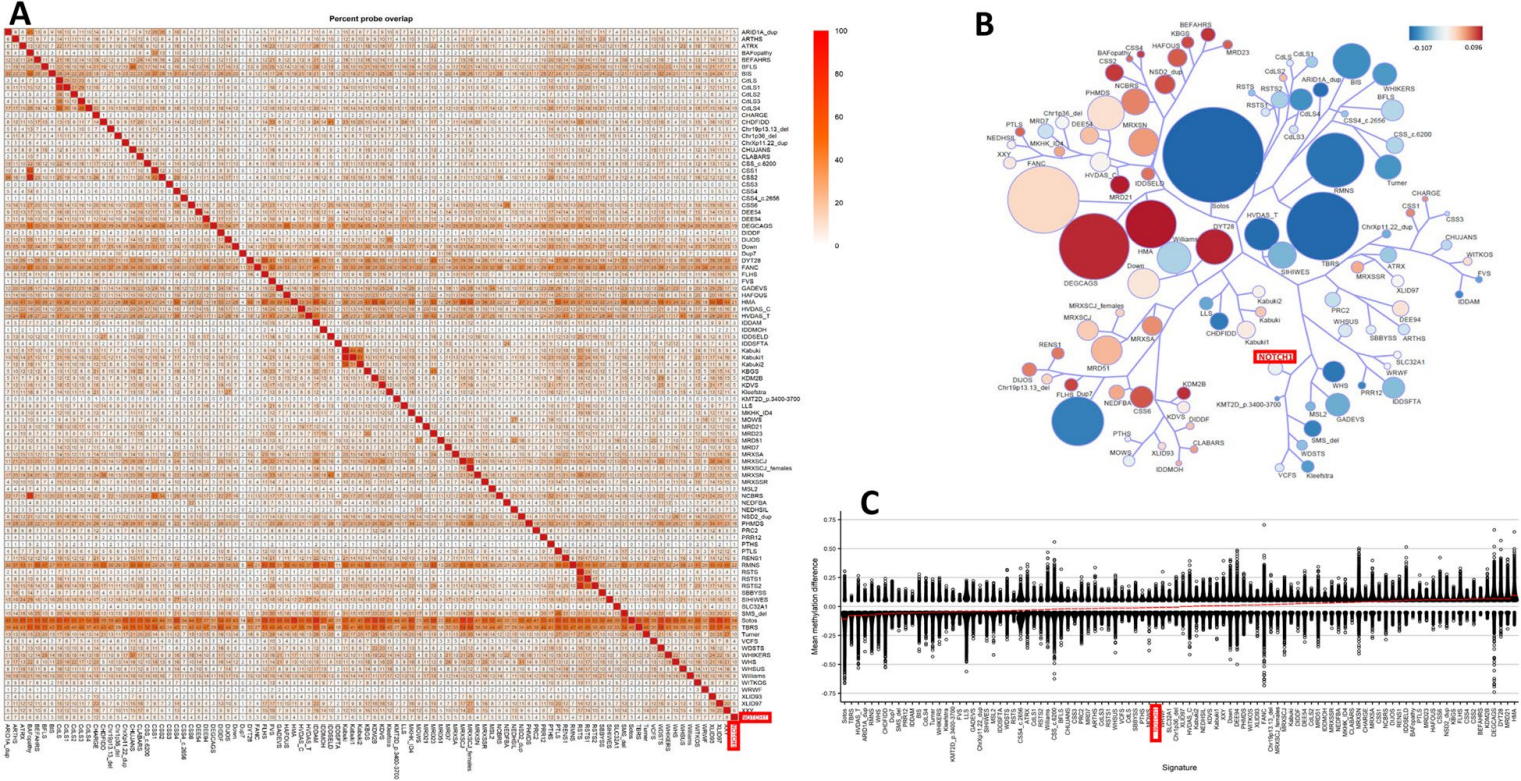
Assessment of the amount of DMPs shared between the NOTCH1 cohort and other syndromes with known episignatures. **A**: Methylation probe overlap. The percentage of DMPs shared between disorders is shown on the colour scale, ranging from white (0%) to red (100%). Each square in the graph represents the percentage of common probes between two syndromes, with the percentage of DMPs from the syndrome on the lower bar that also exist in the DMPs of the syndrome on the right-hand sidebar. **B**: A tree-and-leaf diagram is used, where each node represents a cohort, and syndromes with more similarity in methylation levels are located closer on the tree. Node size is related to the ratio of the number of DMPs to the total number of probes, while node colour demonstrates the overall mean methylation difference in the corresponding cohort. **C**: Comparison of the global mean methylation differences between syndromes with known episignatures

To explore the concurrence between the DNAm profiles defining the *NOTCH1* cohort and those previously identified in 99 other disorders using the EpiSign™ v5 classifier [13, 19, 51, 52], a functional analysis focusing on the overall DNAm alterations observed in the *NOTCH1* cohort was conducted. Regarding the genomic location of the DMPs, most were located within Inter_CGI regions (39%), shores (23%), CDS (40%) and intergenic regions (26%) (Figure S2). Using clustering analyses on the top 500 most significant DMPs for each cohort, the *NOTCH1* cohort exhibited the highest proportion of DMP overlap with Sotos syndrome (34%) and Tatton-Brown-Rahman syndrome (TBRS) (25%) in comparison with 99 other episignatures (Fig. 3A). Furthermore, cluster analysis using tree and leaf plots unveiled similarities between *NOTCH1* and other disorders, notably Lysine Methyltransferase 2D (*KMT2D*_p.3400–3700) and Smith-Magenis syndrome (SMS_del; 17p11.2) groups (Fig. 3B). Finally, the mean differences in β-values between *NOTCH1* and other known episignature disorders revealed more hypomethylation changes in the *NOTCH1* cohort (Fig. 3C).

### Enrichment analysis of probes of the episignature

**Fig. 4:**
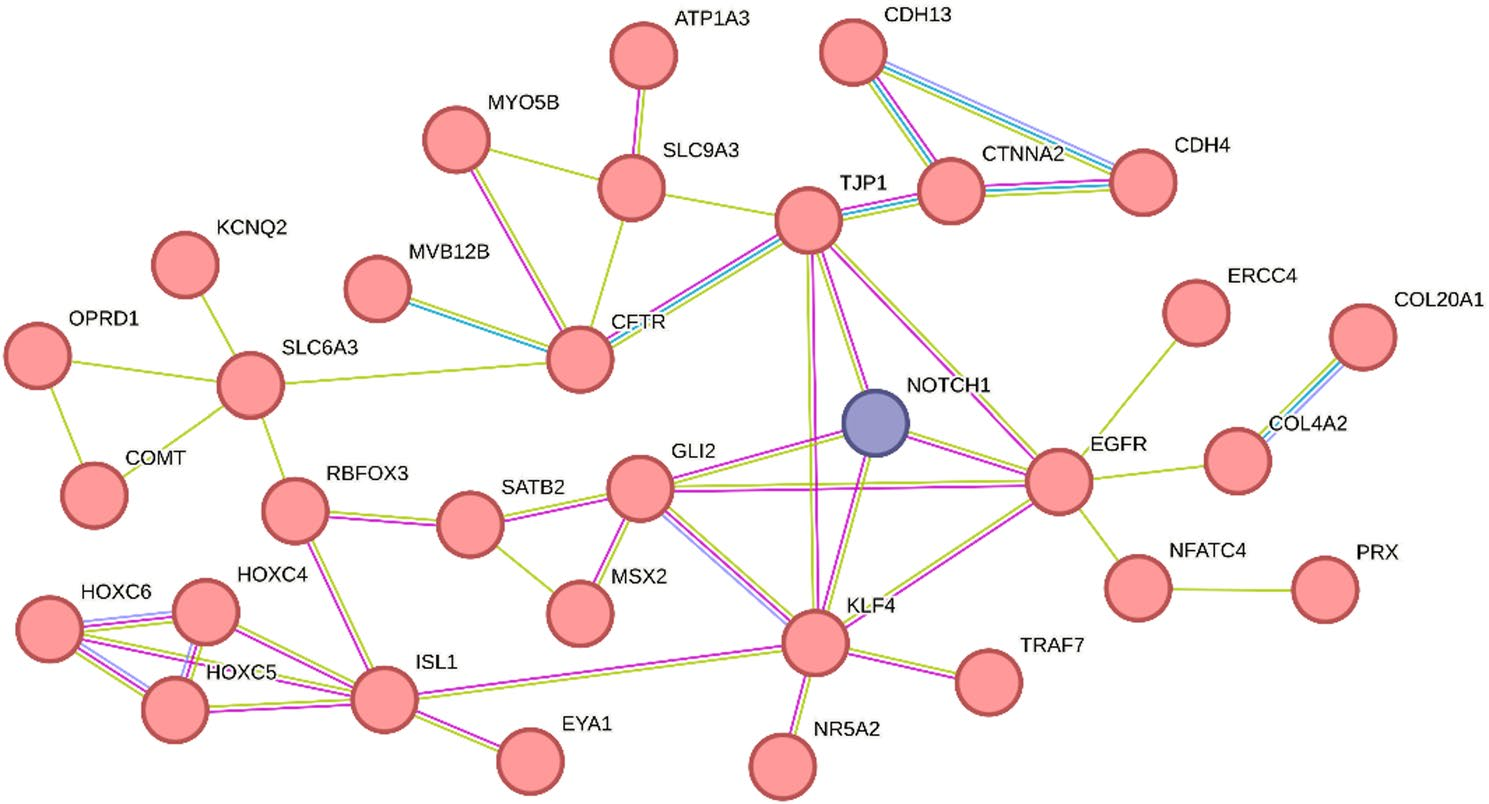
STRING interaction network of genes which are collocated with probes in the ***NOTCH1***-episignature. Nodes represent overlapping genes. Edges represent data indicating an interaction, comprising “Textmining” (green lines), “Experiments” (pink lines) and “Databases” (blue lines). The minimal required interaction score cutoff was defined at >0.4. The full interaction network can be found in Figure S3 [84].

Of the 210 probes contained within the identified episignature, 120 overlap with protein-coding genes (Table S7). Seven of these (*EYA1*,* ISL1*,* MSX2*,* NFATC4*,* PRDM16*,* RAI1* and *TRAF7*) represent genes previously published in the context of cardiac defects [53–59]. Upon performing a STRING analysis of *NOTCH1* with all 120 genes, we noted several interactions, particularly for one large network containing 32 of the 121 genes (Fig. 4, Full String network: Figure S3). Interestingly, GO-term enrichment analysis of the 32 genes of this network revealed “*Regulation of secondary heart field cardioblast proliferation” (GO:0003266)* as the highest-ranking term, involving *NOTCH1*, *ISL1* and *EYA1* (Table S8).

## Discussion

Here we report on a large exome-sequenced cohort, in which we identified ultrarare variants affecting *NOTCH1* as the most common monogenic cause of CHD. In addition, we established a distinct episignature in patients with *NOTCH1*-associated non-syndromic CHD. Given the high abundance of variants in *NOTCH1* in CHD patients, this episignature can possibly contribute to the diagnostic options for these patients.

Our results indicate that deleterious *NOTCH1* variants might account for up to 1% of all CHD cases (38/3907 in our cohort), 2.2% amongst conotruncal defects (22/977) and ToF in particular (17/484; 3.5%). As illustrated by the literature reviewed as part of this work, *NOTCH1* is a well-established contributor to CHD and ToF appears to be one of the predominant cardiac manifestations besides left-sided malformations.

Importantly, for none of our cases with ultra-rare *NOTCH1* variants, was an alternative genetic explanation identified. We also excluded the presence of pathogenic variants in DNA- and histone-methylation related genes, which could possibly interfere with DNA-methylation independently of *NOTCH1*. This further strengthens our conclusion that the episignature correlates to *NOTCH1*-variants.

An interesting finding was the identification of ultra-rare *NOTCH1* variants that specifically impair disulfide bridges in the extracellular region of NOTCH1 in patients with conotruncal defects. This observation is substantiated by previous reports [8, 60, 61]. Disulfide bridges contribute to the correct three-dimensional protein structure and are highly conserved. We hypothesize that alterations of these residues alter the conformation of the extracellular regions, thereby hindering ligand binding and activation of NOTCH1-signalling.

Consequently, specific attention should be paid to cysteine-altering variants when analysing *NOTCH1* variants. Conversely, we also observed four CHD samples with variants that created novel cysteines, while none were found in controls. Novel cysteines might similarly result in an altered protein conformation. Whether this is a relevant disease mechanism remains to be elucidated in further studies.

Of note, an experimentally-derived structure of the full NOTCH1-protein is not available, thus limiting interpretability of structural effects. Nevertheless, we applied in-silico modelling for variants that might potentially alter disulfide-bonding patterns. Not all variants yielded a clear pattern. In some instances, however, modelling information concurred with the methylation signal, providing suggestions for the molecular basis of pathogenicity.

Given the considerable size and clinical importance of the *NOTCH1*-gene, variant assessment is a frequently recurrent task in the diagnostic setting. Indeed, we observed a considerable number of variants with uncertain effects in our cohort.

The mapping of Mendelian disorders with disease-specific DNAm episignature biomarkers and the identification of global disruptions in DNAm profiles are increasingly prevalent [13, 15]. Aberrant DNAm within gene promoters can disturb gene expression and result in abnormal phenotypes [62, 63]. As such episignatures are also extensively employed for the evaluation and reclassification of VUS [15, 63], we investigated whether a differential DNAm episignature is associated with *NOTCH1*-related CHD. We delineate a distinct DNAm episignature and demonstrate that it is sensitive and robust through cross-validation analysis. Additionally, we illustrate the specificity of this signature relative to controls and other episignature disorders.

Episignatures are consistently detectable in peripheral blood across more than 200 genes studied to date, the vast majority of which are associated with conditions lacking any hematologic phenotype. While systematic cross-tissue validation remains an important future research direction, current evidence supports the reliability and clinical utility of peripheral blood–derived episignatures, even though this is not the primary tissue affected in *NOTCH1*-related CHD.

Three cases with ultra-rare NOTCH1 variants (#35, #56, #58), suspected to be pathogenic, clustered with controls and demonstrated an absence of the *NOTCH1-*episignature. Case #35, carrying a variant in a canonical splice acceptor site, displayed an intermediate overlap with the episignature. Exon skipping through disruption of this splice site could potentially preserve the reading frame and result in a shortened, but intact protein. Such an altered protein might still have residual activity, resulting in a hypomorphic effect and generate the intermediate signature overlap. However, due to lack of patient material, we could not validate this using RNA sequencing. Case #56 carried a *de novo* NOTCH1 missense variant (p.(Gly427Arg)). This patient displayed symptoms consistent with Adams-Oliver syndrome, with a comparably minor cardiovascular involvement (patent ductus arteriosus, pulmonary hypertension). Given the phenotype and the poor prediction score for the NOTCH1 signature of this sample, it could indicate that the identified signature might be more informative for severe *NOTCH1*-related cardiac phenotypes, rather than AOS. Lastly, case #58 diagnosed with TGA and carrying the p.(Arg592Cys) NOTCH1 variant, also demonstrated lack of overlap with the *NOTCH1*-episignature. However, this variant was reported in 17 samples in the newest gnomAD freeze (v.4.1.0) and has been listed as benign and as a VUS in ClinVar. Retrospectively, this variant would no longer be considered as (likely) pathogenic. Accordingly, in-silico modelling suggests that this variant is unlikely to result in the formation of a new stable disulfide-bridge.

Epigenetic signatures can be very useful to (re)classify VUS. We therefore investigated four individuals with a *NOTCH1* VUS. Two cases (#15 and #39) clustered with the *NOTCH1-*episignature, indicating that those variants might contribute to disease etiology. Case #15 represents a ToF case with hypothyroidism and renal phenotypes, segregation was not possible due to lack of parental DNA.

For case #39 we could establish paternal inheritance for the VUS. However, to our knowledge, the father does not have CHD or *NOTCH1*-related phenotypes. As incomplete penetrance is frequent in CHD families, analysing the segregation of the episignature in this family would provide valuable insights, as it allows better understanding whether the episignature is a result of altered NOTCH1 activity, or rather indicates a modifying mechanism that enforces a CHD phenotype expression in carriers of *NOTCH1*-variants. Unfortunately, the paternal DNA was not available for methylation testing. Based on in-silico modelling, this variant might lead to a disruption of the hydrophobic environment, increases flexibility of the ANK region, and thereby destabilises MAML1-interaction. Despite the paternal inheritance, both modelling and episignature testing concordantly suggest an effect of this variant. Two cases (#53, #60) did not have overlap with the episignature. Case #53 carrying the p.(Glu242Lys) variant has no cardiac phenotype, but does exhibit various syndromic features. The variant is maternally inherited and the phenotype is also present in the mother, suggesting a possible segregation with the phenotype. The other case (#60), that did not have an overlap with the episignature, carried the p.(Asp622Asn) variant, and comes from a family with pulmonary arterial hypertension. Given the above, although suggestive, we conclude that a negative episignature cannot yet be used as definitive evidence for the absence of pathogenicity. Further analyses, on larger numbers of cases with *NOTCH1* VUS and their epigenetic signatures, are needed to establish this.

While systematic ancestry-focused studies on episignature biomarkers have not been performed, experimental design for feature selection, and available evidence from large scale studies and testing programs supports the robustness of episignatures across ancestral and ethnic backgrounds [19]. Nevertheless, future studies should preferably focus on non-European samples to confirm independence of the episignature from ancestry effects. While some of the cases also presented with extracardiac phenotypes, we did not observe generalizable similarities, especially none for which the episignature might have a predictive value. The current data suggests a limited sensitivity for mild or syndromic cases. Testing additional samples with AOS-features might thus help to assess predictive value outside of nsCHD-cases.

Looking more closely at the differentially methylated positions (DMPs) of the identified *NOTCH1 *signature, we found minimal overlap between the *NOTCH1* signature's DMPs and other established signatures (Fig. 3A), underscoring the highly specific nature of the *NOTCH1* episignature. Sotos- (34%) and TBR-syndrome (25%) had the largest overlaps. Sotos syndrome is caused by heterozygous mutations in the *NSD1* gene and is characterized by overgrowth, facial abnormalities, brain anomalies, seizures, and impaired intellectual development [64]. However, some Sotos patients also present with CHD, which could explain the overlap in some of the DMPs [65]. TBR syndrome is caused by dominant variants in the *DNMT3A* gene and is characterized by impaired intellectual development, face abnormalities, tall stature, seizures, scoliosis and large head circumference. There is only limited literature about patients with TBR syndrome presenting with CHD [66]. 

In addition, the closest established episignatures resembling *NOTCH1* were found to be *KMT2D*-related as well as Smith-Magenis syndrome, which is associated with impairment of *RAI1* (Fig. 3B). Both genes are associated with CHD [67, 68]. Furthermore, one probe of the *NOTCH1*-episignature overlaps with the UTR of *RAI1*.

One important question is how pathogenic *NOTCH1*-variants could generate a specific DNAm-signature. NOTCH-signalling has been reported to interact with histone-methyltransferases such as KDM5A and SETD1A [69, 70]. Moreover, crosstalk between histone-modification and regulation of DNA-methylation patterns is well documented [71]. Given the above, we hypothesize that pathogenic *NOTCH1* variants lead to altered NOTCH1-signalling which in turn affects histone-methyltransferases thereby impacting DNA-methylation patterns. Alternatively, it is known that the NOTCH1 intracellular domain (ICN) localizes to endothelial cell mitochondria, where it enhances mitochondrial metabolism [72]. In addition, a *NOTCH1* variant observed in a non-syndromic ToF patient was demonstrated to decrease ICN mitochondrial localization and pyruvate dehydrogenase activity in heart tissues [72]. Pyruvate dehydrogenase is integral for mitochondrial bioenergetics. This is of interest as recent findings suggest that mitochondrial dysfunction can, in turn, cause alterations in metabolic processes tightly intertwined with DNA methylation, such as the methionine cycle [73]. NOTCH-signalling is one of the earliest and most significant events in (cardiac) development and remains active throughout life. It is therefore conceivable that alterations of this pathway have long lasting implications, amongst others on DNA-methylation. Additional functional testing e.g. applying ChIP-seq and RNA-seq could help shed light on underlying interactions. Due to unavailability of material, these tests could not be integrated in the scope of the presented data.

To look further into the connection between NOTCH1, methylation and CHD we sought to determine whether genes underlying the DMPs of the *NOTCH1*episignature are associated with cardiac development. We identified 120 genes overlapping DMPs, several of these (7/120) are indeed known to be involved in cardiogenesis and have been associated with CHD.

A surprisingly large STRING-interaction network containing 32 genes was found and revealed overrepresentation of the GO-term r*egulation of secondary heart field cardioblast proliferation*. In particular, *ISL1* and *EYA1* emerged as interesting contributors to this process. *ISL1* encodes a transcription factor of the LIM/homeodomain family regulating cell proliferation and survival [74]. It is described as a marker of early progenitor cell populations that contribute to the outflow tract, right ventricle, a subset of left ventricular cells and a large number of atrial cells [75, 76]. Moreover, pathogenic variants in *ISL1* have been reported in patients with CHD (DORV, VSD) [77, 78]. and NOTCH1-signalling has been shown to positively regulate *ISL1*-expression in cardiac progenitor cells [79, 80]. 

*EYA1* is a member of the eyes absent (EYA) family of proteins, which acts as a protein phosphatase and transcriptional coactivator [81]. In humans, variants in *EYA1* are associated with the branchiootorenal syndrome type 1 (OMIM 113650), a condition involving malformations of the ears and kidneys, as well as craniofacial abnormalities. Cardiac abnormalities are typically not part of the spectrum, but double-null *Eya1*-mice display impaired cardiovascular development with an interrupted or right-sided aortic arch, amongst others [82]. Interestingly, the Eya1-Notch1 axis has been shown to play a role in various developmental processes [83]. Dephosphorylation of the intracellular NOTCH1 (ICN) through EYA1 is thought to stabilise the protein, thus contributing to an enhanced NOTCH1-signalling [81]. 

Concluding, genes underlying the DMPs of the *NOTCH1*-episignature have direct and indirect links to (cardiac) development. It is therefore possible that aberrant alterations in methylation of these genes, as a consequence of pathogenic *NOTCH1* variants, could lead to CHD. Future research into these interactions is needed to elucidate the detailed mechanisms.

In general, given the high prevalence of *NOTCH1* variants among patients with CHD and having identified a specific *NOTCH1* DNAm signature, we argue that DNAm analysis can contribute substantially to a more accurate variant assessment, ultimately resulting in improved case management. Moreover, this signature broadens the potential applications of epigenetic testing as DNA from peripheral blood is usually available for individuals undergoing clinical genetic testing. Furthermore, this work can lead to follow-up studies, such as refining sub-signatures amongst the individual subtypes, extending the signature with regards to syndromic NOTCH1-related phenotypes, elucidating the underlying mechanism of this episignature and potentially extending this approach to other genes related to non-syndromic CHD.

## Conclusions

In this work we report on one of the largest cohorts with *NOTCH1*-associated CHD cases. Our analysis identified variants disrupting disulfide-bonds as a novel and frequent mechanism for conotruncal malformations. Overall, deleterious variants in *NOTCH1* are found in 1% of CHD cases and over 2% of conotruncal malformations, making it the most common monogenic cause in this type of disorder. In addition, we established a *NOTCH1*-specific DNAm-signature, representing the first such signature in non-syndromic CHD-cases. We also show that genes underlying this signature have direct and indirect links to (cardiac) development and CHD. Overall, this novel signature considerably broadens the applicability of epigenetic testing and facilitates assessment of *NOTCH1* variant pathogenicity.

## Supplementary Information

Additional file 1: Supplementary figures S1 to S9 including cross validation plots, annotations of differentially methylated probes, full STRING interaction network for genes overlapping the presented signature, a NOTCH1-protein plot representing the DNAm prediction scores for tested samples and figures depicting the results of in-silico modelling for cysteine-altering variants.

Additional file 2: Supplementary tables S1 to S8 containing domains and modification site annotations, GO-enrichment results, published NOTCH1-variants, details on the in-silico modelling, an overview of disulfide-bond residues in NOTCH1 as well as prediction scores and probe information for the NOTCH1-episignature. 

## Data Availability

Datasets used in this study that are publicly available are described in Am J Hum Genet. 2020;106(3):356–70.(19) Anonymized data for each subject from that dataset are described in the study. Individual genomic, epigenomic, or other personally identifiable data for other samples in the EpiSign Knowledge Database (EKD) cannot be deposited in publicly accessible databases due to institutional and ethics restrictions. These include data and samples submitted from external institutions to the London Health Sciences Centre EKD under Institutional Material and Data Transfer Agreements, data submitted for episignature assessment under Research Services Agreements, and research study cohorts under Institutional Research Ethics Board approvals (Western University REB 106302 and REB 116108). Some software packages used in this study are publicly available as described in the Materials and Methods section. EpiSign™ is a commercial software and is not publicly available.The CRAM-level data from CHD patients used in this study can be accessed under 675 the following accession codes (European Genome-phenome Archive): 676 EGAD00001002200, EGAD00001000796, EGAD00001000797, EGAD00001000800, 677 EGAS00001000544, EGAS00001000775, EGAS00001000762. UK Biobank 50 K 678 WES dataset freeze was accessed under the application number 44165.

## References

[1] Hoffman JIE, Kaplan S. The incidence of congenital heart disease. J Am Coll Cardiol. 2002;39(12):1890-900. 10.1016/S0735-1097(02)01886-7. 10.1016/s0735-1097(02)01886-7 12084585

[2] van der Linde D, Konings EEM, Slager MA, Witsenburg M, Helbing WA, Takkenberg JJM, et al. Birth prevalence of congenital heart disease worldwide. J Am Coll Cardiol. 2011;58(21):2241–7. 22078432 10.1016/j.jacc.2011.08.025

[3] Øyen N, Poulsen G, Boyd HA, Wohlfahrt J, Jensen PKA, Melbye M. National time trends in congenital heart defects, Denmark, 1977–2005. Am Heart J. 2009;157(3):467–e4731. 19249416 10.1016/j.ahj.2008.10.017

[4] Pierpont ME, Brueckner M, Chung WK, et al. Genetic Basis for Congenital Heart Disease: Revisited: A Scientific Statement From the American Heart Association. Circulation. 2018;138(21):e653-e711. 10.1161/CIR.0000000000000606, https://pubmed.ncbi.nlm.nih.gov/30571578/. 10.1161/CIR.0000000000000606 PMC6555769 30571578

[5] MacGrogan D, Münch J, De La Pompa JL. Notch and interacting signalling pathways in cardiac development, disease, and regeneration. Nat Rev Cardiol. 2018;15(11):685–704. 30287945 10.1038/s41569-018-0100-2

[6] Hatakeyama J, Kageyama R. Notch1 expression is spatiotemporally correlated with neurogenesis and negatively regulated by Notch1-independent Hes genes in the developing nervous system. Cereb Cortex. 2006;16(suppl1):i132-7. 16766699 10.1093/cercor/bhj166

[7] Kerstjens-Frederikse WS, van de Laar IMBH, Vos YJ, Verhagen JMA, Berger RMF, Lichtenbelt KD, et al. Cardiovascular malformations caused by NOTCH1 mutations do not keep left: data on 428 probands with left-sided CHD and their families. Genet Med. 2016;18(9):914–23. 26820064 10.1038/gim.2015.193

[8] Page DJ, Miossec MJ, Williams SG, Monaghan RM, Fotiou E, Cordell HJ, et al. Whole exome sequencing reveals the major genetic contributors to nonsyndromic tetralogy of fallot. Circ Res. 2019;124(4):553–63. 30582441 10.1161/CIRCRESAHA.118.313250 PMC6377791

[9] Loscalzo ML, Goh DLM, Loeys B, Kent KC, Spevak PJ, Dietz HC. Familial thoracic aortic dilation and bicommissural aortic valve: a prospective analysis of natural history and inheritance. Am J Med Genet A. 2007;143A(17):1960–7. 17676603 10.1002/ajmg.a.31872

[10] Weng AP, Ferrando AA, Lee W, Morris JP, Silverman LB, Sanchez-Irizarry C, et al. Activating mutations of *NOTCH1* in human T cell acute lymphoblastic leukemia. Science. 2004;306(5694):269–71. 15472075 10.1126/science.1102160

[11] Reedijk M, Odorcic S, Chang L, Zhang H, Miller N, McCready DR, et al. High-level coexpression of *JAG1* and *NOTCH1* is observed in human breast cancer and is associated with poor overall survival. Cancer Res. 2005;65(18):8530–7. 16166334 10.1158/0008-5472.CAN-05-1069

[12] Lehman A, Wuyts W, Patel MS. Adams-Oliver Syndrome In: Adam MP, Bick S, Mirzaa GM, Pagon RA, Wallace SE, Amemiya A, eds. GeneReviews®. Seattle (WA): University of Washington, Seattle; April 14, 2016. https://pubmed.ncbi.nlm.nih.gov/27077170/.

[13] Levy MA, McConkey H, Kerkhof J, Barat-Houari M, Bargiacchi S, Biamino E, et al. Novel diagnostic DNA methylation episignatures expand and refine the epigenetic landscapes of Mendelian disorders. Hum Genet Genomics Adv. 2022;3(1):100075. 10.1016/j.xhgg.2021.100075 PMC8756545 35047860

[14] Sadikovic B, Levy MA, Kerkhof J, Aref-Eshghi E, Schenkel L, Stuart A, et al. Clinical epigenomics: genome-wide DNA methylation analysis for the diagnosis of Mendelian disorders. Genet Med. 2021;23(6):1065–74. 33547396 10.1038/s41436-020-01096-4 PMC8187150

[15] Aref-Eshghi E, Bend EG, Colaiacovo S, Caudle M, Chakrabarti R, Napier M, et al. Diagnostic utility of genome-wide DNA methylation testing in genetically unsolved individuals with suspected hereditary conditions. Am J Hum Genet. 2019;104(4):685–700. 30929737 10.1016/j.ajhg.2019.03.008 PMC6451739

[16] Kerkhof J, Squeo GM, McConkey H, Levy MA, Piemontese MR, Castori M, et al. DNA methylation episignature testing improves molecular diagnosis of Mendelian chromatinopathies. Genet Med. 2022;24(1):51–60. 34906459 10.1016/j.gim.2021.08.007

[17] Aref-Eshghi E, Kerkhof J, Pedro VP, Groupe DI, France, Barat-Houari M, Ruiz-Pallares N, et al. Evaluation of DNA methylation episignatures for diagnosis and phenotype correlations in 42 Mendelian neurodevelopmental disorders. Am J Hum Genet. 2020;106(3):356–70. 32109418 10.1016/j.ajhg.2020.01.019 PMC7058829

[18] Aref-Eshghi E, Rodenhiser DI, Schenkel LC, Lin H, Skinner C, Ainsworth P, et al. Genomic DNA methylation signatures enable concurrent diagnosis and clinical genetic variant classification in neurodevelopmental syndromes. Am J Hum Genet. 2018;102(1):156–74. 29304373 10.1016/j.ajhg.2017.12.008 PMC5777983

[19] Kerkhof J, Rastin C, Levy MA, Relator R, McConkey H, Demain L, et al. Diagnostic utility and reporting recommendations for clinical DNA methylation episignature testing in genetically undiagnosed rare diseases. Genet Med. 2024;26(5):101075. 38251460 10.1016/j.gim.2024.101075

[20] Zaidi S, Choi M, Wakimoto H, Ma L, Jiang J, Overton JD, et al. De novo mutations in histone-modifying genes in congenital heart disease. Nature. 2013;498(7453):220–3. 23665959 10.1038/nature12141 PMC3706629

[21] Lim TB, Foo SYR, Chen CK. The role of epigenetics in congenital heart disease. Genes. 2021;12(3):390. 33803261 10.3390/genes12030390 PMC7998561

[22] Gong J, Sheng W, Ma D, Huang G, Liu F. DNA methylation status of TBX20 in patients with tetralogy of fallot. BMC Med Genomics. 2019;12(1):75. 31138201 10.1186/s12920-019-0534-3 PMC6540552

[23] Sifrim A, Hitz MP, Wilsdon A, Breckpot J, Turki SHA, Thienpont B, et al. Distinct genetic architectures for syndromic and nonsyndromic congenital heart defects identified by exome sequencing. Nat Genet. 2016;48(9):1060–5. 27479907 10.1038/ng.3627 PMC5988037

[24] Audain E, Wilsdon A, Breckpot J, Izarzugaza JMG, Fitzgerald TW, Kahlert AK, et al. Integrative analysis of genomic variants reveals new associations of candidate haploinsufficient genes with congenital heart disease. PLoS Genet. 2021;17(7):e1009679. 34324492 10.1371/journal.pgen.1009679 PMC8354477

[25] McLaren W, Gil L, Hunt SE, Riat HS, Ritchie GRS, Thormann A, et al. The ensembl variant effect predictor. Genome Biol. 2016. . 10.1186/s13059-016-0974-4 27268795 10.1186/s13059-016-0974-4 PMC4893825

[26] Richards S, Aziz N, Bale S, Bick D, Das S, Gastier-Foster J, et al. Standards and guidelines for the interpretation of sequence variants: a joint consensus recommendation of the American college of medical genetics and genomics and the association for molecular pathology. Genet Med. 2015;17(5):405–24. 25741868 10.1038/gim.2015.30 PMC4544753

[27] Ellard S, Baple EL, Callaway A, Berry I, Forrester N, Turnbull C et al. ACGS Best Practice Guidelines for Variant Classification in Rare Disease 2020. 2020. https://www.acgs.uk.com/media/11631/uk-practice-guidelines-for-variant-classification-v4-01-2020.pdf.

[28] Rentzsch P, Witten D, Cooper GM, Shendure J, Kircher M. CADD: predicting the deleteriousness of variants throughout the human genome. Nucleic Acids Res. 2018;47(D1):D886–94. 10.1093/nar/gky1016 PMC6323892 30371827

[29] Ioannidis NM, Rothstein JH, Pejaver V, Middha S, McDonnell SK, Baheti S, et al. REVEL: an ensemble method for predicting the pathogenicity of rare missense variants. Am J Hum Genet. 2016;99(4):877–85. 27666373 10.1016/j.ajhg.2016.08.016 PMC5065685

[30] Samocha KE, Kosmicki JA, Karczewski KJ, O’Donnell-Luria AH, Pierce-Hoffman E, MacArthur DG et al. Regional missense constraint improves variant deleteriousness prediction [Internet]. Genomics; 2017 Jun [Cited 2023 May 26]. Available from: http://biorxiv.org/lookup/doi/10.1101/148353

[31] Pejaver V, Byrne AB, Feng BJ, Pagel KA, Mooney SD, Karchin R, et al. Calibration of computational tools for missense variant pathogenicity classification and ClinGen recommendations for PP3/BP4 criteria. Am J Hum Genet. 2022;109(12):2163–77. 36413997 10.1016/j.ajhg.2022.10.013 PMC9748256

[32] Jaganathan K, Kyriazopoulou Panagiotopoulou S, McRae JF, Darbandi SF, Knowles D, Li YI, et al. Predicting splicing from primary sequence with deep learning. Cell. 2019;176(3):535–e54824. 30661751 10.1016/j.cell.2018.12.015

[33] Vander Velde ET, Vriend JWJ, Mannens MMAM, Uiterwaal CSPM, Brand R, Mulder BJM. CONCOR, an initiative towards a National registry and DNA-bank of patients with congenital heart disease in the netherlands: rationale, design, and first results. Eur J Epidemiol. 2005;20(6):549–57. 16121765 10.1007/s10654-005-4264-9

[34] Lesurf R, Breckpot J, Bouwmeester J, Hanafi N, Jain A, Liang Y, et al. A validated heart-specific model for splice-disrupting variants in childhood heart disease. Genome Med. 2024;16(1):119. 39402625 10.1186/s13073-024-01383-8 PMC11476204

[35] Bolger AM, Lohse M, Usadel B. Trimmomatic: a flexible trimmer for illumina sequence data. Bioinformatics. 2014;30(15):2114–20. 24695404 10.1093/bioinformatics/btu170 PMC4103590

[36] Li H, Durbin R. Fast and accurate short read alignment with Burrows-Wheeler transform. Bioinforma Oxf Engl. 2009;25(14):1754–60. 10.1093/bioinformatics/btp324 PMC2705234 19451168

[37] Danecek P, Bonfield JK, Liddle J, Marshall J, Ohan V, Pollard MO, et al. Twelve years of samtools and BCFtools. Gigascience. 2021;10(2):giab008. 33590861 10.1093/gigascience/giab008 PMC7931819

[38] Tan A, Abecasis GR, Kang HM. Unified representation of genetic variants. Bioinformatics. 2015;31(13):2202–4. 25701572 10.1093/bioinformatics/btv112 PMC4481842

[39] Pedersen BS, Layer RM, Quinlan AR. Vcfanno: fast, flexible annotation of genetic variants. Genome Biol. 2016;17(1):118. 27250555 10.1186/s13059-016-0973-5 PMC4888505

[40] Aryee MJ, Jaffe AE, Corrada-Bravo H, Ladd-Acosta C, Feinberg AP, Hansen KD, et al. Minfi: a flexible and comprehensive bioconductor package for the analysis of infinium DNA methylation microarrays. Bioinformatics. 2014;30(10):1363–9. 24478339 10.1093/bioinformatics/btu049 PMC4016708

[41] Ho DE, Imai K, King G, Stuart EA. MatchIt: Nonparametric Preprocessing for Parametric Causal Inference. J Stat Softw . 2011 [Cited 2024 Jun 20];42(8). Available from: http://www.jstatsoft.org/v42/i08/

[42] Ritchie ME, Phipson B, Wu D, Hu Y, Law CW, Shi W, et al. Limma powers differential expression analyses for RNA-sequencing and microarray studies. Nucleic Acids Res. 2015;43(7):e47–47. 25605792 10.1093/nar/gkv007 PMC4402510

[43] Levy MA, Relator R, McConkey H, Pranckeviciene E, Kerkhof J, Barat-Houari M, et al. Functional correlation of genome‐wide DNA methylation profiles in genetic neurodevelopmental disorders. Hum Mutat. 2022;43(11):1609–28. 35904121 10.1002/humu.24446

[44] Gu Z, Gu L, Eils R, Schlesner M, Brors B. Circlize implements and enhances circular visualization in R. Bioinformatics. 2014;30(19):2811–2. 24930139 10.1093/bioinformatics/btu393

[45] Cardoso MA, Rizzardi LEA, Kume LW, Groeneveld CS, Trefflich S, Morais DAA, et al. TreeAndLeaf: an R/Bioconductor package for graphs and trees with focus on the leaves. Bioinformatics. 2022;38(5):1463–4. 34864914 10.1093/bioinformatics/btab819

[46] Cavalcante RG, Sartor MA. annotatr: genomic regions in context. Valencia A, editor. Bioinformatics. 2017;33(15):2381–3. 28369316 10.1093/bioinformatics/btx183 PMC5860117

[47] Leaver-Fay A, Tyka M, Lewis SM, Lange OF, Thompson J, Jacak R, et al. ROSETTA3: an object-oriented software suite for the simulation and design of macromolecules. Methods Enzymol. 2011;487:545–74. 21187238 10.1016/B978-0-12-381270-4.00019-6 PMC4083816

[48] Chaudhury S, Lyskov S, Gray JJ. PyRosetta: a script-based interface for implementing molecular modeling algorithms using Rosetta. Bioinforma Oxf Engl. 2010;26(5):689–91. 10.1093/bioinformatics/btq007 PMC2828115 20061306

[49] Tyka MD, Keedy DA, André I, Dimaio F, Song Y, Richardson DC, et al. Alternate states of proteins revealed by detailed energy landscape mapping. J Mol Biol. 2011;405(2):607–18. 21073878 10.1016/j.jmb.2010.11.008 PMC3046547

[50] Alford RF, Leaver-Fay A, Jeliazkov JR, O’Meara MJ, DiMaio FP, Park H, et al. The rosetta all-atom energy function for macromolecular modeling and design. J Chem Theory Comput. 2017;13(6):3031–48. 28430426 10.1021/acs.jctc.7b00125 PMC5717763

[51] Aref-Eshghi E, Schenkel LC, Lin H, Skinner C, Ainsworth P, Paré G, et al. Clinical validation of a genome-wide DNA methylation assay for molecular diagnosis of imprinting disorders. J Mol Diagn. 2017;19(6):848–56. 28807811 10.1016/j.jmoldx.2017.07.002

[52] Bjornsson HT. The mendelian disorders of the epigenetic machinery. Genome Res. 2015;25(10):1473–81. 26430157 10.1101/gr.190629.115 PMC4579332

[53] Li B, Xu L, Hong N, Chen S, Xu R. In silico analyses reveal the relationship between SIX1/EYA1 mutations and conotruncal heart defects. Pediatr Cardiol. 2018;39(1):176–82. 29043394 10.1007/s00246-017-1744-0

[54] Osoegawa K, Schultz K, Yun K, Mohammed N, Shaw GM, Lammer EJ. Haploinsufficiency of *insulin gene enhancer protein 1* (*ISL1*) is associated with d-transposition of the great arteries. Mol Genet Genomic Med. 2014;2(4):341–51. 25077177 10.1002/mgg3.75 PMC4113275

[55] Chen YH, Ishii M, Sun J, Sucov HM, Maxson RE. Msx1 and Msx2 regulate survival of secondary heart field precursors and post-migratory proliferation of cardiac neural crest in the outflow tract. Dev Biol. 2007;308(2):421–37. 17601530 10.1016/j.ydbio.2007.05.037

[56] Bushdid PB, Osinska H, Waclaw RR, Molkentin JD, Yutzey KE. NFATc3 and NFATc4 are required for cardiac development and mitochondrial function. Circ Res. 2003;92(12):1305–13. 12750314 10.1161/01.RES.0000077045.84609.9F

[57] Arndt AK, Schafer S, Drenckhahn JD, Sabeh MK, Plovie ER, Caliebe A, et al. Fine mapping of the 1p36 deletion syndrome identifies mutation of PRDM16 as a cause of cardiomyopathy. Am J Hum Genet. 2013;93(1):67–77. 23768516 10.1016/j.ajhg.2013.05.015 PMC3710750

[58] Girirajan S, Vlangos CN, Szomju BB, Edelman E, Trevors CD, Dupuis L, et al. Genotype-phenotype correlation in Smith-Magenis syndrome: evidence that multiple genes in 17p11.2 contribute to the clinical spectrum. Genet Med Off J Am Coll Med Genet. 2006;8(7):417–27. 10.1097/01.gim.0000228215.32110.89 16845274

[59] Tokita MJ, Chen CA, Chitayat D, Macnamara E, Rosenfeld JA, Hanchard N, et al. De novo missense variants in TRAF7 cause developmental delay, congenital anomalies, and dysmorphic features. Am J Hum Genet. 2018;103(1):154–62. 29961569 10.1016/j.ajhg.2018.06.005 PMC6035372

[60] Chapman G, Moreau JLM, Eddie IP, Szot JO, Iyer KR, Shi H, et al. Functional genomics and gene-environment interaction highlight the complexity of congenital heart disease caused by Notch pathway variants. Hum Mol Genet. 2020;29(4):566–79. 31813956 10.1093/hmg/ddz270 PMC7068028

[61] Yaoita H, Kawai E, Takayama J, Iwasawa S, Saijo N, Abiko M, et al. Genetic etiology of truncus arteriosus excluding 22q11.2 deletion syndrome and identification of c.1617del, a prevalent variant in TMEM260, in the Japanese population. J Hum Genet. 2024;69(5):177–83. 38351237 10.1038/s10038-024-01223-y PMC11043042

[62] Der Laan LV, Rooney K, Trooster TM, Mannens MM, Sadikovic B, Henneman P. DNA methylation episignatures: insight into copy number variation. Epigenomics. 2022;14(21):1373–88. 36537268 10.2217/epi-2022-0287

[63] Sadikovic B, Aref-Eshghi E, Levy MA, Rodenhiser D. DNA methylation signatures in Mendelian developmental disorders as a diagnostic bridge between genotype and phenotype. Epigenomics. 2019;11(5):563–75. 30875234 10.2217/epi-2018-0192

[64] Kurotaki N, Imaizumi K, Harada N, Masuno M, Kondoh T, Nagai T, et al. Haploinsufficiency of NSD1 causes sotos syndrome. Nat Genet. 2002;30(4):365–6. 11896389 10.1038/ng863

[65] Calcagni G, Ferrigno F, Franceschini A, Dentici ML, Capolino R, Sinibaldi L, et al. Congenital heart defects in patients with molecularly confirmed sotos syndrome. Diagnostics. 2024;14(6):594. 38535015 10.3390/diagnostics14060594 PMC10968944

[66] Ghaoui R, Ha TT, Kerkhof J, McConkey H, Gao S, Babic M, et al. Expanding the phenotype of DNMT3A as a cause a congenital myopathy with rhabdomyolysis. Neuromuscul Disord. 2023;33(6):484–9. 37209493 10.1016/j.nmd.2023.04.002

[67] Onesimo R, Versacci P, Delogu AB, De Rosa G, Pugnaloni F, Blandino R, et al. Smith-Magenis syndrome: report of morphological and new functional cardiac findings with review of the literature. Am J Med Genet A. 2021;185(7):2003–11. 33811726 10.1002/ajmg.a.62196

[68] Digilio MC, Gnazzo M, Lepri F, Dentici ML, Pisaneschi E, Baban A, et al. Congenital heart defects in molecularly proven Kabuki syndrome patients. Am J Med Genet A. 2017;173(11):2912–22. 28884922 10.1002/ajmg.a.38417

[69] Liefke R, Oswald F, Alvarado C, Ferres-Marco D, Mittler G, Rodriguez P, et al. Histone demethylase KDM5A is an integral part of the core Notch–RBP-J repressor complex. Genes Dev. 2010;24(6):590–601. 20231316 10.1101/gad.563210 PMC2841336

[70] Chai H, Pan C, Zhang M, Huo H, Shan H, Wu J. Histone methyltransferase SETD1A interacts with Notch and promotes Notch transactivation to augment ovarian cancer development. BMC Cancer. 2023;23(1):96. 36707804 10.1186/s12885-023-10573-3 PMC9883963

[71] Tibben BM, Rothbart SB. Mechanisms of DNA methylation regulatory function and crosstalk with histone lysine methylation. J Mol Biol. 2024;436(7):168394. 38092287 10.1016/j.jmb.2023.168394 PMC10957332

[72] Wang J, Zhao R, Xu S, Zhou XY, Cai K, Chen YL, et al. NOTCH1 mitochondria localization during heart development promotes mitochondrial metabolism and the endothelial-to-mesenchymal transition in mice. Nat Commun. 2024;15(1):9945. 39550366 10.1038/s41467-024-54407-7 PMC11569218

[73] Lopes FC. Mitochondrial metabolism and DNA methylation: a review of the interaction between two genomes. Clin Epigenetics. 2020;12(1):182. 33228792 10.1186/s13148-020-00976-5 PMC7684747

[74] Golzio C, Havis E, Daubas P, Nuel G, Babarit C, Munnich A, et al. ISL1 directly regulates FGF10 transcription during human cardiac outflow formation. PLoS One. 2012;7(1):e30677. 22303449 10.1371/journal.pone.0030677 PMC3267757

[75] Pandur P, Sirbu IO, Kühl SJ, Philipp M, Kühl M. Islet1-expressing cardiac progenitor cells: a comparison across species. Dev Genes Evol. 2013;223(1–2):117–29. 22526874 10.1007/s00427-012-0400-1 PMC3552366

[76] Ren J, Miao D, Li Y, Gao R. Spotlight on Isl1: a key player in cardiovascular development and diseases. Front Cell Dev Biol. 2021;9:793605. 34901033 10.3389/fcell.2021.793605 PMC8656156

[77] Wang Z, Song HM, Wang F, Zhao CM, Huang RT, Xue S, et al. A new *ISL1* loss-of-function mutation predisposes to congenital double outlet right ventricle. Int Heart J. 2019;60(5):1113–22. 31484864 10.1536/ihj.18-685

[78] Ma L, Wang J, Li L, Qiao Q, Di RM, Li XM, et al. ISL1 loss-of-function mutation contributes to congenital heart defects. Heart Vessels. 2019;34(4):658–68. 30390123 10.1007/s00380-018-1289-z

[79] Jiao J, Tian W, Qiu P, Norton EL, Wang MM, Chen YE, et al. Induced pluripotent stem cells with NOTCH1 gene mutation show impaired differentiation into smooth muscle and endothelial cells: implications for bicuspid aortic valve-related aortopathy. J Thorac Cardiovasc Surg. 2018;156(2):515–e5221. 29653750 10.1016/j.jtcvs.2018.02.087 PMC9809054

[80] Kwon C, Qian L, Cheng P, Nigam V, Arnold J, Srivastava D. A regulatory pathway involving Notch1/beta-catenin/Isl1 determines cardiac progenitor cell fate. Nat Cell Biol. 2009;11(8):951–7. 19620969 10.1038/ncb1906 PMC2748816

[81] Soni UK, Roychoudhury K, Hegde RS. The eyes absent proteins in development and in developmental disorders. Biochem Soc Trans. 2021;49(3):1397–408. 34196366 10.1042/BST20201302 PMC8286820

[82] Fraser FC, Aymé S, Halal F, Sproule J, Opitz JM. Autosomal dominant duplication of the renal collecting system, hearing loss, and external ear anomalies: a new syndrome? Am J Med Genet. 1983;14(3):473–8. 6859100 10.1002/ajmg.1320140311

[83] Zhang H, Wang L, Wong EYM, Tsang SL, Xu PX, Lendahl U, et al. An Eya1-Notch axis specifies bipotential epibranchial differentiation in mammalian craniofacial morphogenesis. eLife. 2017;6:e30126. 29140246 10.7554/eLife.30126 PMC5705218

[84] Szklarczyk D, Kirsch R, Koutrouli M, Nastou K, Mehryary F, Hachilif R, et al. The STRING database in 2023: protein-protein association networks and functional enrichment analyses for any sequenced genome of interest. Nucleic Acids Res. 2023;51(D1):D638–46. 36370105 10.1093/nar/gkac1000 PMC9825434

