# Supplementary material for: DNA methylation analysis of NOTCH1 variants reveals the first episignature for non-syndromic congenital heart defects
Source: Genome Med. 2026 Jan 7;18:2. doi: 10.1186/s13073-025-01587-6 (PMC12781588; doi:10.1186/s13073-025-01587-6)
Supplement: Supplementary file 2 — Additional file 2: Supplementary tables S1 to S8 containing domains and modification site annotations, GO-enrichment results, published NOTCH1-variants, details on the in-silico modelling, an overview of disulfide-bond residues in NOTCH1 as well as prediction scores and probe information for the NOTCH1-episignature. [file 13073_2025_1587_MOESM2_ESM.pptx]

## Slide 1
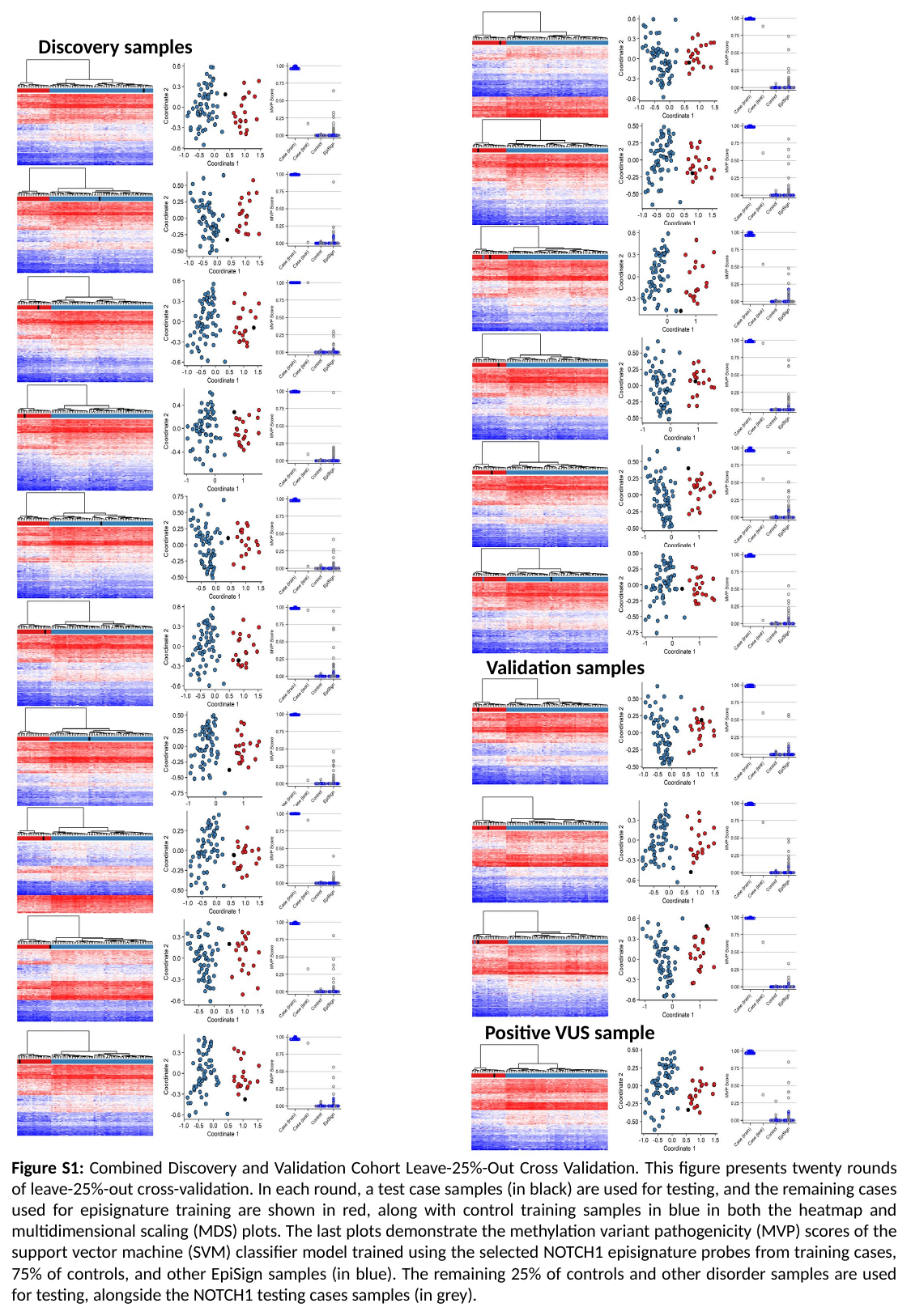

Discovery samples
Validation samples
Positive VUS sample
Figure S1: Combined Discovery and Validation Cohort Leave-25%-Out Cross Validation. This figure presents twenty rounds of leave-25%-out cross-validation. In each round, a test case samples (in black) are used for testing, and the remaining cases used for episignature training are shown in red, along with control training samples in blue in both the heatmap and multidimensional scaling (MDS) plots. The last plots demonstrate the methylation variant pathogenicity (MVP) scores of the support vector machine (SVM) classifier model trained using the selected NOTCH1 episignature probes from training cases, 75% of controls, and other EpiSign samples (in blue). The remaining 25% of controls and other disorder samples are used for testing, alongside the NOTCH1 testing cases samples (in grey).

## Slide 2
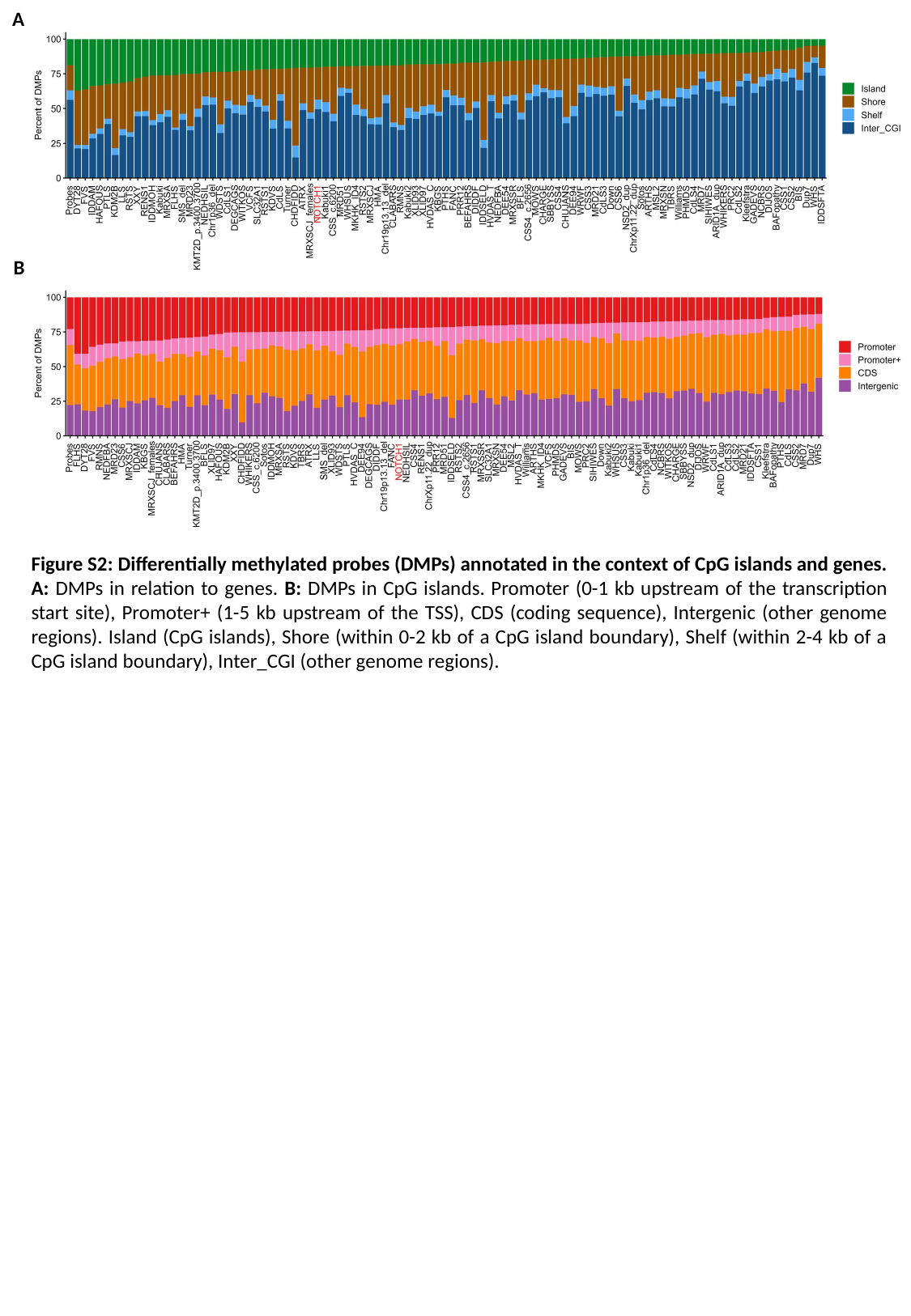

A
B
Figure S2: Differentially methylated probes (DMPs) annotated in the context of CpG islands and genes. A: DMPs in relation to genes. B: DMPs in CpG islands. Promoter (0-1 kb upstream of the transcription start site), Promoter+ (1-5 kb upstream of the TSS), CDS (coding sequence), Intergenic (other genome regions). Island (CpG islands), Shore (within 0-2 kb of a CpG island boundary), Shelf (within 2-4 kb of a CpG island boundary), Inter_CGI (other genome regions).

## Slide 3
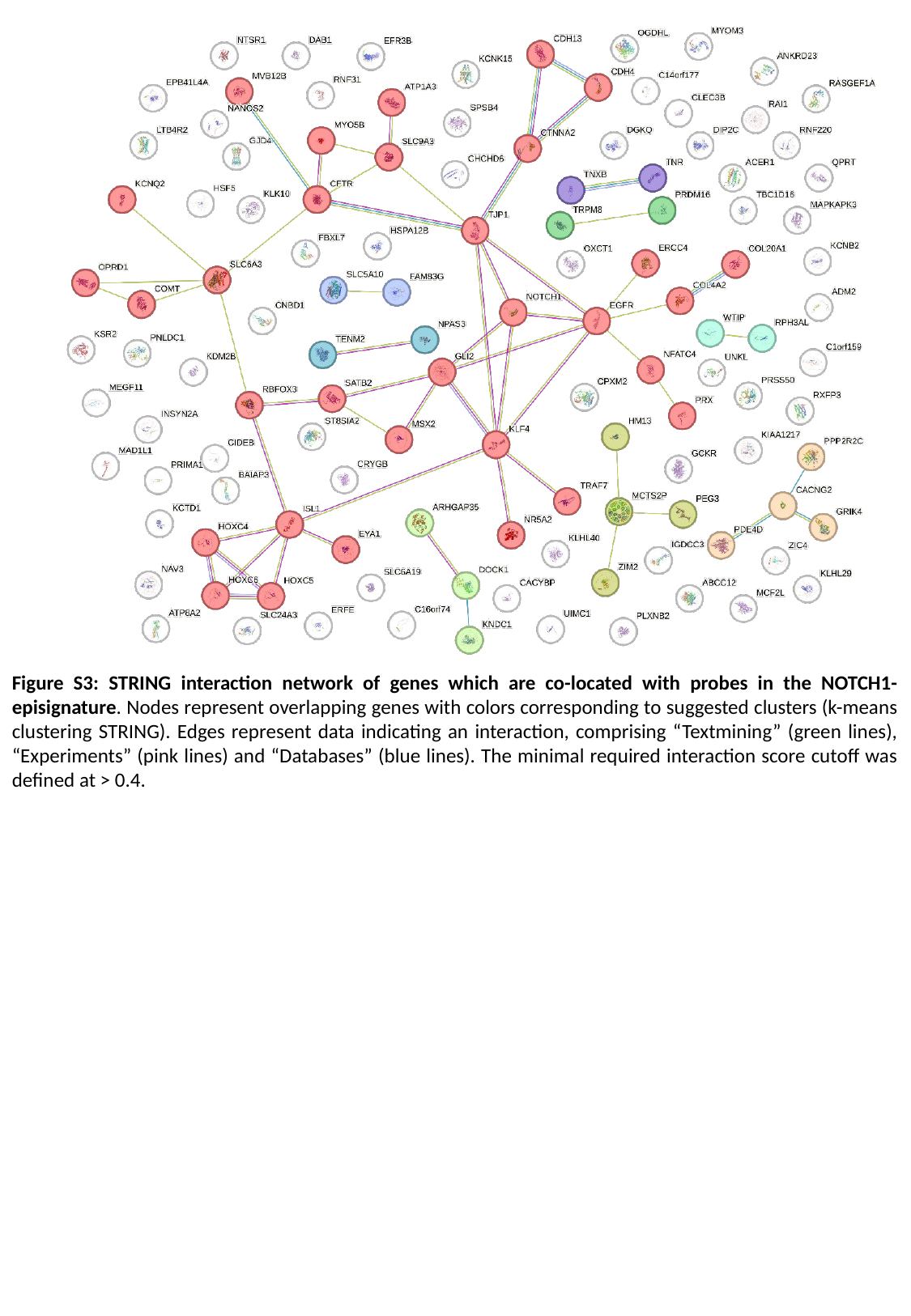

Figure S3: STRING interaction network of genes which are co-located with probes in the NOTCH1-episignature. Nodes represent overlapping genes with colors corresponding to suggested clusters (k-means clustering STRING). Edges represent data indicating an interaction, comprising “Textmining” (green lines), “Experiments” (pink lines) and “Databases” (blue lines). The minimal required interaction score cutoff was defined at > 0.4.

## Slide 4
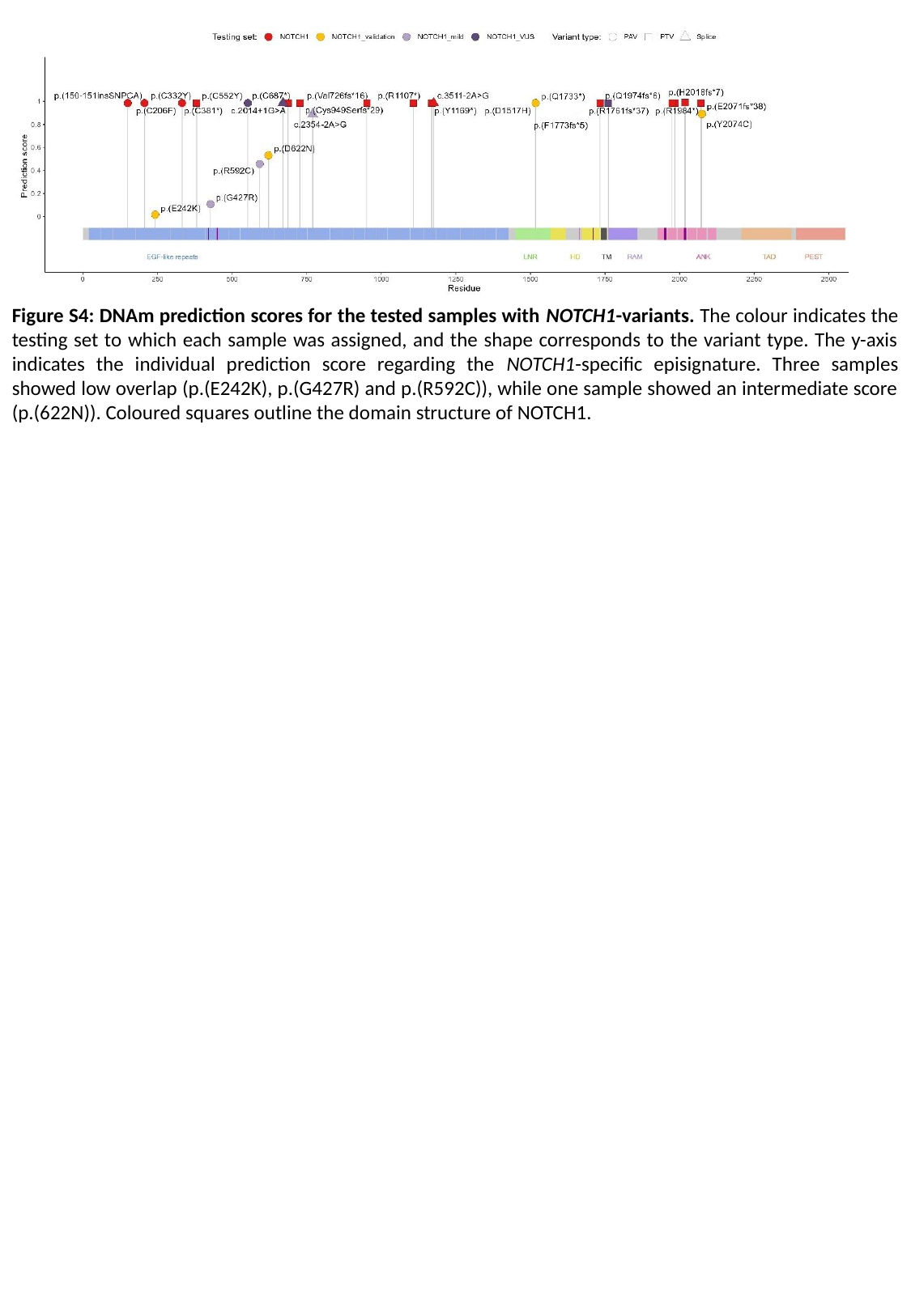

Figure S4: DNAm prediction scores for the tested samples with NOTCH1-variants. The colour indicates the testing set to which each sample was assigned, and the shape corresponds to the variant type. The y-axis indicates the individual prediction score regarding the NOTCH1-specific episignature. Three samples showed low overlap (p.(E242K), p.(G427R) and p.(R592C)), while one sample showed an intermediate score (p.(622N)). Coloured squares outline the domain structure of NOTCH1.

## Slide 5
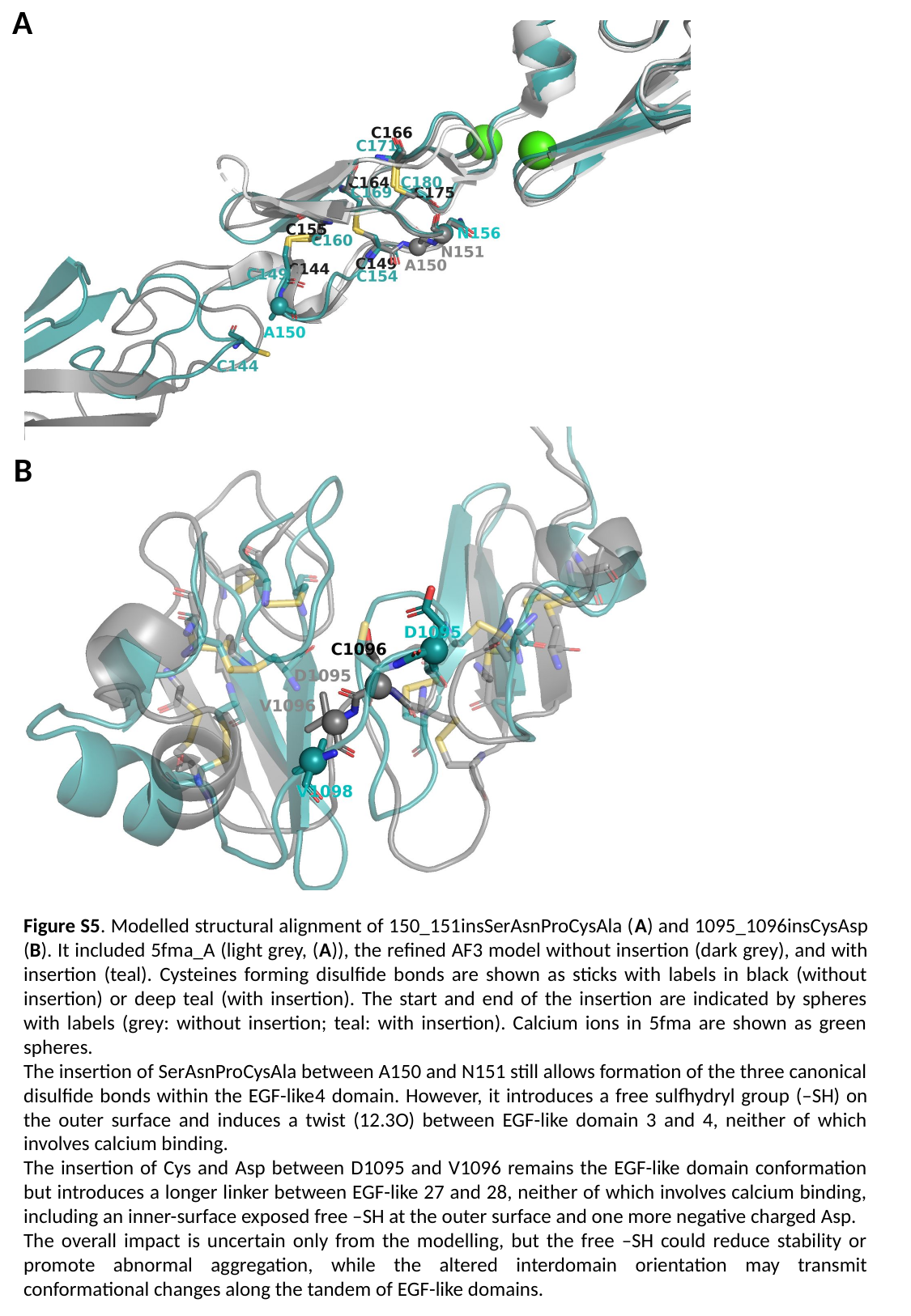

A
Figure S5. Modelled structural alignment of 150_151insSerAsnProCysAla (A) and 1095_1096insCysAsp (B). It included 5fma_A (light grey, (A)), the refined AF3 model without insertion (dark grey), and with insertion (teal). Cysteines forming disulfide bonds are shown as sticks with labels in black (without insertion) or deep teal (with insertion). The start and end of the insertion are indicated by spheres with labels (grey: without insertion; teal: with insertion). Calcium ions in 5fma are shown as green spheres.
The insertion of SerAsnProCysAla between A150 and N151 still allows formation of the three canonical disulfide bonds within the EGF-like4 domain. However, it introduces a free sulfhydryl group (–SH) on the outer surface and induces a twist (12.3O) between EGF-like domain 3 and 4, neither of which involves calcium binding.
The insertion of Cys and Asp between D1095 and V1096 remains the EGF-like domain conformation but introduces a longer linker between EGF-like 27 and 28, neither of which involves calcium binding, including an inner-surface exposed free –SH at the outer surface and one more negative charged Asp.
The overall impact is uncertain only from the modelling, but the free –SH could reduce stability or promote abnormal aggregation, while the altered interdomain orientation may transmit conformational changes along the tandem of EGF-like domains.
B

## Slide 6
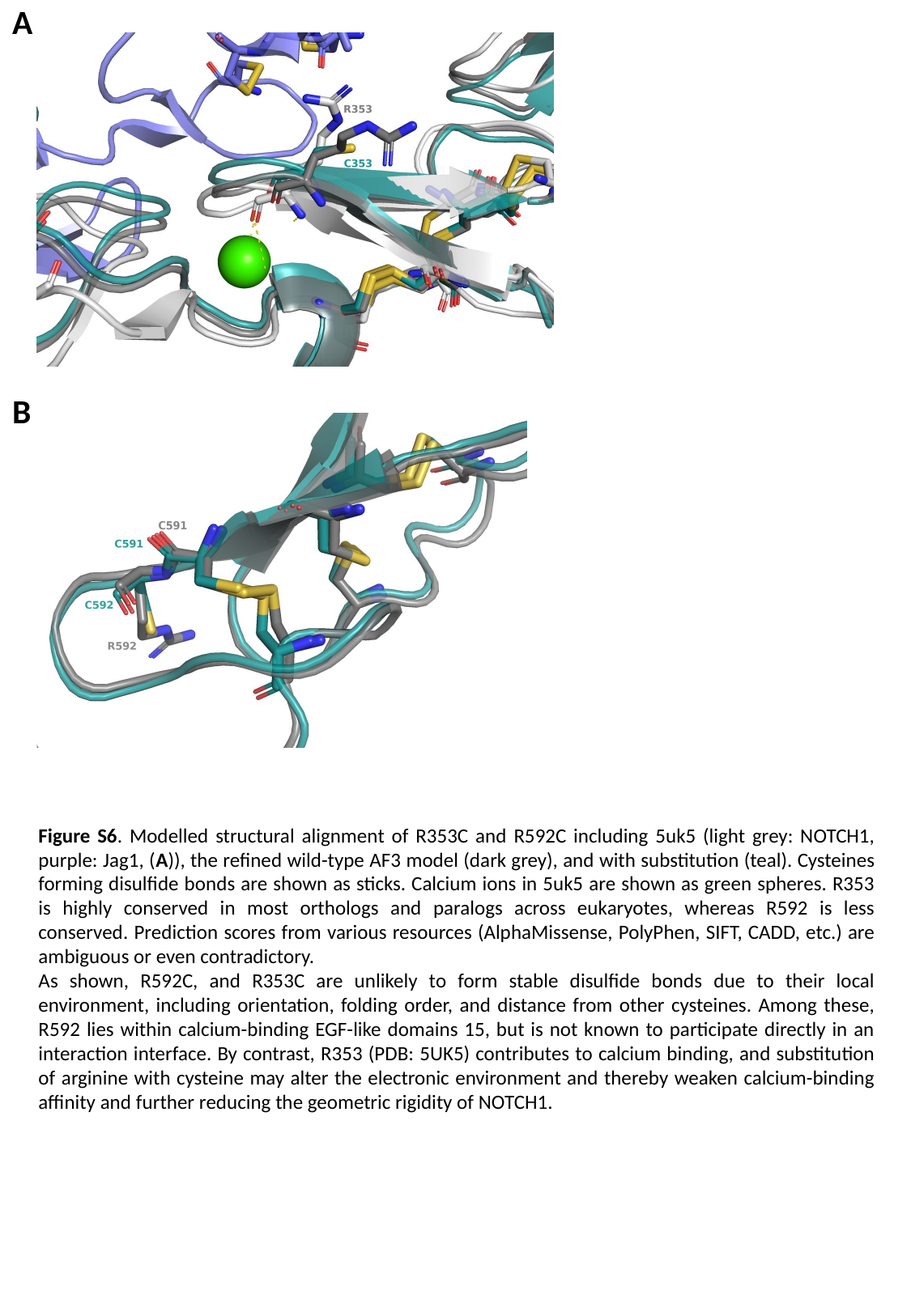

A
Figure S6. Modelled structural alignment of R353C and R592C including 5uk5 (light grey: NOTCH1, purple: Jag1, (A)), the refined wild-type AF3 model (dark grey), and with substitution (teal). Cysteines forming disulfide bonds are shown as sticks. Calcium ions in 5uk5 are shown as green spheres. R353 is highly conserved in most orthologs and paralogs across eukaryotes, whereas R592 is less conserved. Prediction scores from various resources (AlphaMissense, PolyPhen, SIFT, CADD, etc.) are ambiguous or even contradictory.
As shown, R592C, and R353C are unlikely to form stable disulfide bonds due to their local environment, including orientation, folding order, and distance from other cysteines. Among these, R592 lies within calcium-binding EGF-like domains 15, but is not known to participate directly in an interaction interface. By contrast, R353 (PDB: 5UK5) contributes to calcium binding, and substitution of arginine with cysteine may alter the electronic environment and thereby weaken calcium-binding affinity and further reducing the geometric rigidity of NOTCH1.
B

## Slide 7
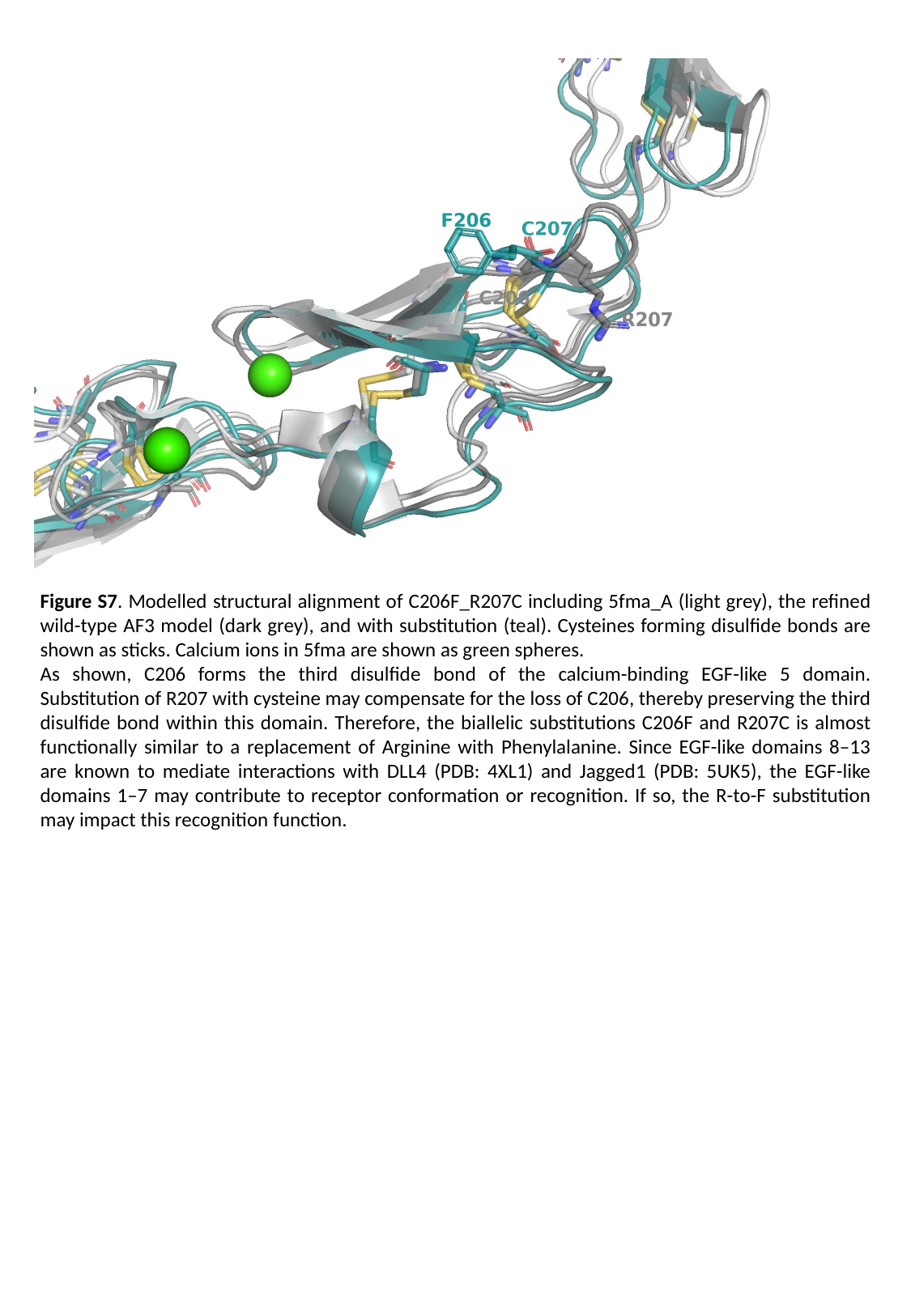

Figure S7. Modelled structural alignment of C206F_R207C including 5fma_A (light grey), the refined wild-type AF3 model (dark grey), and with substitution (teal). Cysteines forming disulfide bonds are shown as sticks. Calcium ions in 5fma are shown as green spheres.
As shown, C206 forms the third disulfide bond of the calcium-binding EGF-like 5 domain. Substitution of R207 with cysteine may compensate for the loss of C206, thereby preserving the third disulfide bond within this domain. Therefore, the biallelic substitutions C206F and R207C is almost functionally similar to a replacement of Arginine with Phenylalanine. Since EGF-like domains 8–13 are known to mediate interactions with DLL4 (PDB: 4XL1) and Jagged1 (PDB: 5UK5), the EGF-like domains 1–7 may contribute to receptor conformation or recognition. If so, the R-to-F substitution may impact this recognition function.

## Slide 8
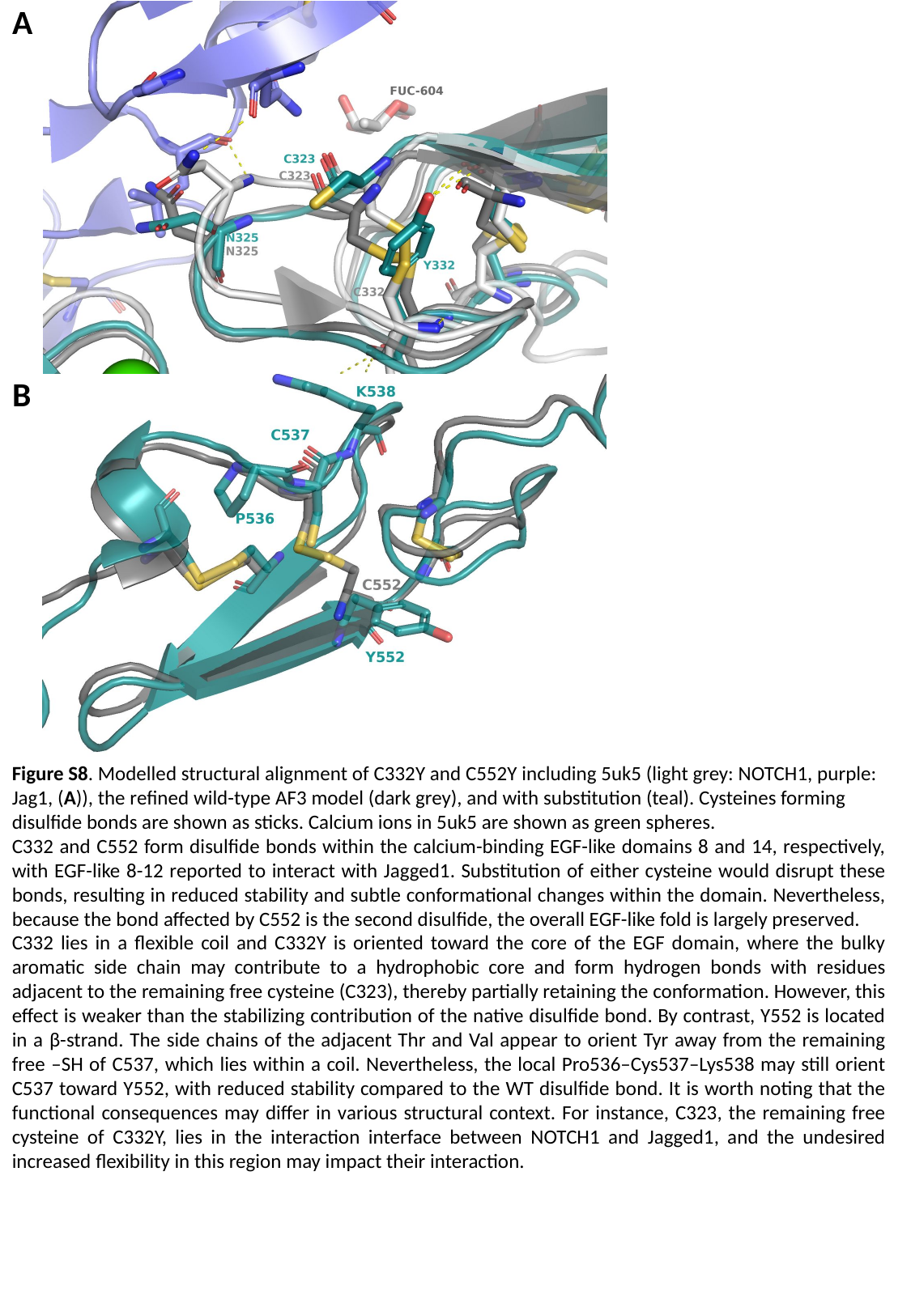

A
Figure S8. Modelled structural alignment of C332Y and C552Y including 5uk5 (light grey: NOTCH1, purple: Jag1, (A)), the refined wild-type AF3 model (dark grey), and with substitution (teal). Cysteines forming disulfide bonds are shown as sticks. Calcium ions in 5uk5 are shown as green spheres.
C332 and C552 form disulfide bonds within the calcium-binding EGF-like domains 8 and 14, respectively, with EGF-like 8-12 reported to interact with Jagged1. Substitution of either cysteine would disrupt these bonds, resulting in reduced stability and subtle conformational changes within the domain. Nevertheless, because the bond affected by C552 is the second disulfide, the overall EGF-like fold is largely preserved.
C332 lies in a flexible coil and C332Y is oriented toward the core of the EGF domain, where the bulky aromatic side chain may contribute to a hydrophobic core and form hydrogen bonds with residues adjacent to the remaining free cysteine (C323), thereby partially retaining the conformation. However, this effect is weaker than the stabilizing contribution of the native disulfide bond. By contrast, Y552 is located in a β-strand. The side chains of the adjacent Thr and Val appear to orient Tyr away from the remaining free –SH of C537, which lies within a coil. Nevertheless, the local Pro536–Cys537–Lys538 may still orient C537 toward Y552, with reduced stability compared to the WT disulfide bond. It is worth noting that the functional consequences may differ in various structural context. For instance, C323, the remaining free cysteine of C332Y, lies in the interaction interface between NOTCH1 and Jagged1, and the undesired increased flexibility in this region may impact their interaction.
B

## Slide 9
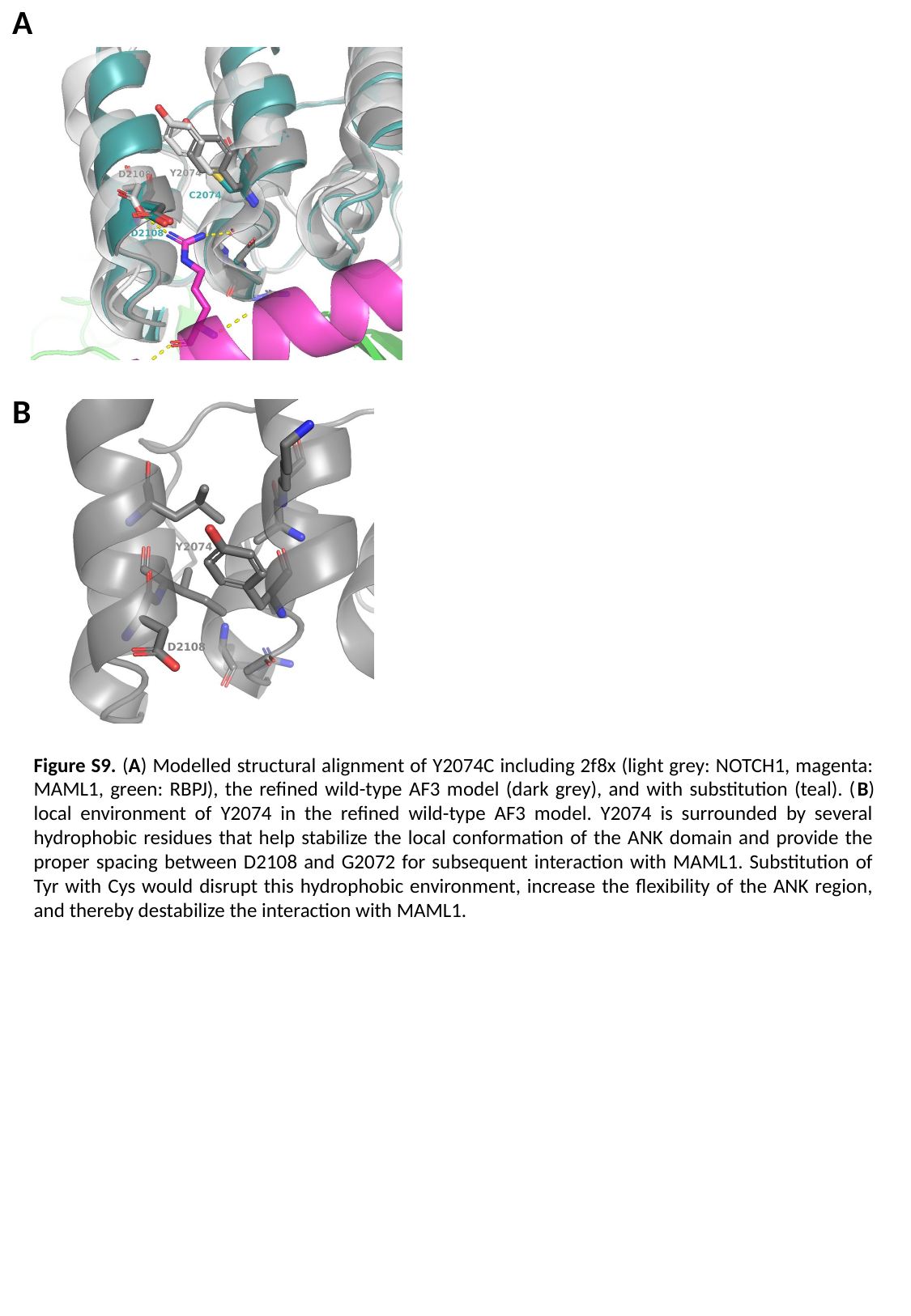

A
B
Figure S9. (A) Modelled structural alignment of Y2074C including 2f8x (light grey: NOTCH1, magenta: MAML1, green: RBPJ), the refined wild-type AF3 model (dark grey), and with substitution (teal). (B) local environment of Y2074 in the refined wild-type AF3 model. Y2074 is surrounded by several hydrophobic residues that help stabilize the local conformation of the ANK domain and provide the proper spacing between D2108 and G2072 for subsequent interaction with MAML1. Substitution of Tyr with Cys would disrupt this hydrophobic environment, increase the flexibility of the ANK region, and thereby destabilize the interaction with MAML1.
